# Genome-Wide Identification of Evolutionarily Conserved Alternative Splicing Events in Flowering Plants

**DOI:** 10.3389/fbioe.2015.00033

**Published:** 2015-03-26

**Authors:** Srikar Chamala, Guanqiao Feng, Carolina Chavarro, W. Brad Barbazuk

**Affiliations:** ^1^Department of Biology, University of Florida, Gainesville, FL, USA; ^2^Graduate Program in Plant Molecular and Cellular Biology, University of Florida, Gainesville, FL, USA; ^3^Center for Applied Genetic Technologies, University of Georgia, Athens, GA, USA; ^4^Genetics Institute, University of Florida, Gainesville, FL, USA

**Keywords:** alternative spicing, plants, comparative genomics, RNA-seq, transcriptome

## Abstract

Alternative splicing (AS) plays important roles in many plant functions, but its conservation across the plant kingdom is not known. We describe a methodology to identify AS events and identify conserved AS events across large phylogenetic distances using RNA-Seq datasets. We applied this methodology to transcriptome data from nine angiosperms including *Amborella*, the single sister species to all other extant flowering plants. AS events within 40–70% of the expressed multi-exonic genes per species were found, 27,120 of which are conserved among two or more of the taxa studied. While many events are species specific, many others are shared across long evolutionary distances suggesting they have functional significance. Conservation of AS event data provides an estimate of the number of ancestral AS events present at each node of the tree representing the nine species studied. Furthermore, the presence or absence of AS isoforms between species with different whole genome duplication (WGD) histories provides the opportunity to examine the impact of WDG on AS potential. Examining AS in gene families identifies those with high rates of AS, and conservation can distinguish ancient events vs. recent or species specific adaptations. The MADS-box and SR protein families are found to represent families with low and high occurrences of AS, respectively, yet their AS events were likely present in the MRCA of angiosperms.

## Introduction

Alternative splicing (AS) is a post-transcriptional modification of precursor mRNA (pre-mRNA) that can result in the formation of multiple distinct mRNAs from a single gene. This process is one mechanism through which eukaryotes generate transcriptome and proteome diversity, and can also play a role in regulating protein abundance (Reddy, 2007; Barbazuk et al., 2008). There is evidence that AS plays critical roles in many essential plant functions such as photosynthesis, defense response, flowering, and cereal grain quality (Barbazuk et al., 2008). Despite the important roles AS plays in plants, the evolution and conservation of AS events across plant species is not well understood. This is largely due to lack of abundant transcriptome sequence data sampled from multiple and comparable tissues across diverse flowering plants (Reddy, 2007; Barbazuk et al., 2008). Most large-scale, cross-species, global-scale AS comparisons in plants have been limited to identifying conserved AS events using cDNA and expressed sequence tag (EST) sequences, and these comparative studies in plants reported few conserved events between species (Wang and Brendel, 2006; Baek et al., 2008; Wang et al., 2008; Severing et al., 2009). A recent study comparing *Brassica* and *Arabidopsis* identified many more conserved AS events, i.e., 537 AS events in 485 genes (Darracq and Adams, 2013); likely the result of deeper sequence data sets. However, the results of these studies still underestimate AS in plants since they do not examine transcriptome diversity in all tissues (Darracq and Adams, 2013). High-throughput, deep sequencing technologies, and multi-tissue sampling increase estimates of the frequency of AS events (Syed et al., 2012). The last few years have seen the addition of whole genome and transcriptome sequence collections for many plants that span broad evolutionary distances. These resources allow the study of genome-wide AS event conservation and evolution in plants. Discovery of conserved events across phylogenetically diverse organisms implies a likely biological relevance and identifies AS isoforms that may perform essential roles (Reddy, 2007; Barbazuk et al., 2008).

In addition to identifying conserved AS events between plants, understanding where whole genome duplication (WGDs) events have occurred throughout angiosperm lineages (Soltis et al., 2009; Jiao et al., 2011; Vanneste et al., 2014) enables one to investigate changes in AS associated with WGD. In spite of this, only one study in *Arabidopsis thaliana* by Zhang et al. (2010) has investigated the evolutionary conservation and divergence of AS patterns in genes duplicated by polyploidy events. This study was limited in scope by only examining AS events within 52 WGD duplicate *Arabidopsis* gene pairs previously reported by Wang and Brendel (2006), who also reported that only 20% of genes in *Arabidopsis* undergo AS, while recent reports identify AS in over 60% of *Arabidopsis* genes (Marquez et al., 2012).

In this study, we investigated the conservation of AS patterns in genes across angiosperm lineages and examine this data in light of lineage specific and/or clade restricted polyploidy events that have taken place during angiosperm evolution. We developed a computational framework that identifies and classifies AS events from publicly available whole genome draft sequences and their corresponding high throughput, deep transcriptome sequence data sets available in the public domain, and identifies AS event conservation across the species examined. Using this framework, we identified AS events genome wide within the legume model systems: common bean (*Phaseolus vulgaris*) and soybean (*Glycine max*). Common bean and soybean are the two most closely related species within our study, having diverged about 19 MYA (Lavin et al., 2005). After their divergence, the soybean underwent a lineage-specific WGD about 5–10 MYA (Schmutz et al., 2010; Roulin et al., 2012). Thus, soybean and common bean provide a model system for the examination of conserved AS events between soybean and common bean, enabling examination of the direct impact of WGD on AS. We investigated AS changes in expressed multi-exonic genes from 14,759 gene sets (Schmutz et al., 2014), where single gene in common bean have two orthologs that resulted from soybean’s recent WGD. Interestingly, even though common bean and soybean diverged only 19 MYA, merely 35% of the detected AS events in common bean have conservation in at least one co-ortholog in soybean, suggesting that most events are lineage-specific. A similar trend is also found among AS events between co-orthologs of soybean that diverged about 10 MYA, where only 28% events are conserved.

Based on our success in identifying conserved AS events between common bean and soybean, we extended this analysis to include nine angiosperm taxa distributed across the angiosperm phylogenetic tree constituting seven eudicots, one monocot (*Oryza sativa* – rice), and *Amborella trichopoda* (Amborella Genome Project, 2013; Chamala et al., 2013), a pivotal species that is sister to all other angiosperms. Our software pipeline identified AS events across all nine species with the proportion of expressed multi-exonic genes that exhibited alternative splicing ranging from 40 to 70%, as well as conserved events between all possible combinations of the nine species surveyed. The size of conserved AS event collections range from a maximum of 5,202 conserved events identified between common bean and soybean, to minimum of 101, which constitutes AS events conserved across all nine species. Overall, our pipeline has identified thousands of candidates AS events, some of which have been conserved broadly across long evolutionary distances, and these data provide many interesting candidate genes for future functional studies.

One outcome of identifying conserved AS events is the ability to examine AS conservation and changes among members of gene families within a single species, or across multiple species. Such data can reveal gene families that experience higher rates of AS than others – as seen in the Serine/Arginine protein family (Richardson et al., 2011). To illustrate this point, AS in the important MADS-box and SR protein families were closely examined in this study. Members of MADS-box transcription factor gene family are involved in controlling major aspects of the life of land plants (Gramzow and Theissen, 2010) and are well-known for regulation of floral organ development (Causier et al., 2010). SR proteins function in spliceosome assembly, and participate in regulating constitutive and AS of pre-mRNAs, including their own transcripts (Richardson et al., 2011). The proportion of genes exhibiting AS is low within the members of the MADS-box gene family type, somewhat higher in MADS-box type II genes, and very high in SR protein gene families. Similar trends were seen in cross-species AS event conservation with 0, 5, and 34 AS events being conserved within sub-families of MADS-box type I, MADS-box type II, and SR protein gene families, respectively.

Additionally, identifying conserved AS events can inform on the impact gene duplication has had on maintaining or creating new AS events. Genomes, genes, and gene families are thought to change through the processes of fractionation, sub- and neo-functionalization following a WGD event. It’s possible that following WGD, these same processes may also impact the AS potential of a given loci and lead to loss and/or acquisition of isoforms at a given loci. Indeed, there are reports of sub-functionalization of AS isoforms within genes of plants that have resulted in the fixation of separate isoforms into separate members of a parologous gene pair both (Cusack and Wolfe, 2007; Rösti and Denyer, 2007). In this same light, there is some evidence from model animal systems that large gene families, presumably produced through gene duplication, exhibit less AS among their members than seen within members of small gene families or singleton genes (Moore and Purugganan, 2005; Ober, 2005; Su et al., 2006; Su and Gu, 2012). Because most plants have undergone multiple WGD events during their evolutionary history, it is possible that this could have decreased abundance of AS in plants overall, and most dramatically within plant lineages that have undergone multiple rounds of WGD.

## Materials and Methods

### Genomic and transcriptomic data collection

#### Genome assemblies and annotations

Genome assemblies and protein coding gene annotations for Amborella (Version 1.0) and Medicago (Version Mt4.0v1) were obtained from http://amborella.org and http://www.jcvi.org/medicago, respectively, genome assemblies and annotation for the remaining seven taxa studied were obtained from Phytozome v9.0 (Goodstein et al., 2012). Supplementary Table 1 summarizes the sources of genome assemblies and annotations along with basic gene annotation metrics. Only protein-coding genes with at least one intron were used in downstream analysis.

#### Transcriptome collection

All transcriptome data (Supplementary Table 2) (including ESTs, full and partial mRNA sequence, and RNA-seq), were collected from both public and private resources as listed in Supplementary Table 3.

### RNA-seq data processing and assembly

**Figure 1:**
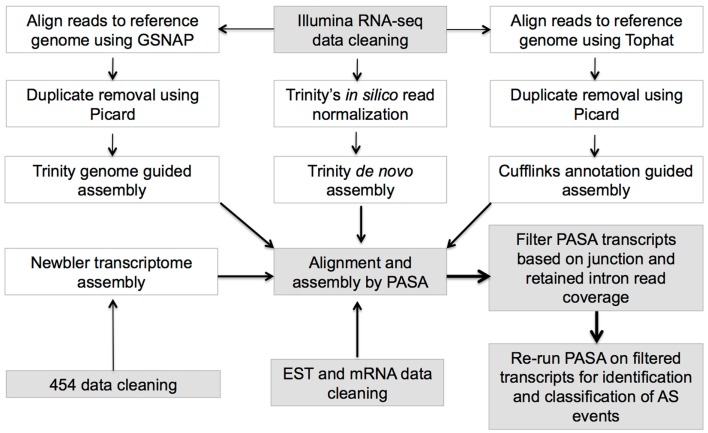
**Workflow of transcriptome data pre-processing and PASA assembly**.

Three different methodologies involving PASA (Haas et al., 2003), Trinity genome-guided^1^, and Trinity *de novo* transcriptome assemblers (Haas et al., 2013) were implemented for assembling RNA-seq data (Figure 1; Supplementary Table 3) to maximize the recovery of all possible isoforms (for details, see Supplementary Methods 1.1).

### PASA pipeline

EST, mRNA, and RNA-seq assemblies (above) were run through PASA 2.0 (Haas et al., 2003), which performs a splice-aware alignment to a reference genome, builds transcript assemblies from the alignments by identifying unique assemblies and collapsing redundant models, and identifies and characterizes AS events. The following parameters were used for running the PASA pipeline: cufflinks_gtf, C, R, g, t, T, u, CPU 5, ALT_SPLICE, “ALIGNER blat,gmap,” INVALIDATE_SINGLE_EXON_ESTS, and MAX_INTRON_LENGTH (which is the same as the 99th percentile intron sizes, Supplementary Table 4). By default, PASA only keeps near-perfect transcript alignments with at least 95% identity and covering at least 90% of the transcript length (Campbell et al., 2006). Transcripts were discarded if one or more junctions were not supported by a minimum of two reads, or in the case of intron retention isoforms, the retained intron region must have at least median read coverage of two. AS events defined by PASA were processed through an in-house software pipeline to identify and re-classify those AS events that contained both alternative 5′ and 3′ splice sites simultaneously.

### OrthoMCL clustering

The OrthoMCL pipeline (Li et al., 2003) was used with standard settings to identify potential orthologous gene families (orthogroups) between species listed in Supplementary Table 1 using protein sequences from the longest isoform of each gene as input. Orthogroups resulting from OrthoMCL were reported in Supplementary Data 1.

### Identification of conserved AS events between taxa

**Figure 2:**
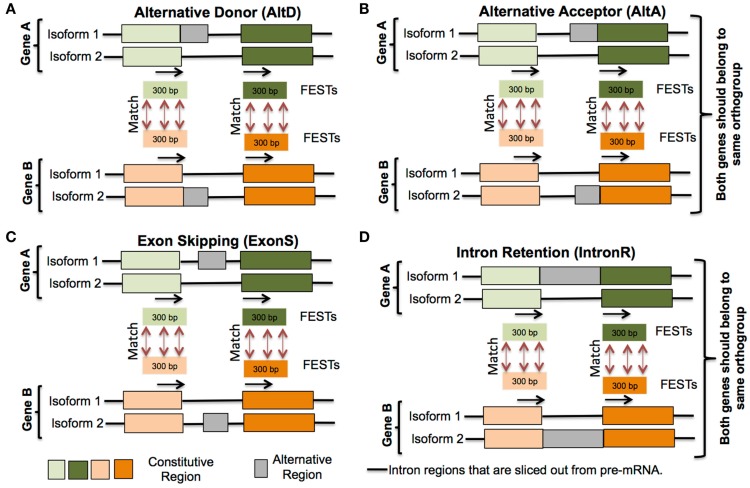
**Conserved alternative splicing events identification pipeline**. (i) Extract 30–300 bp flanking exon sequence tags (FESTs) at alternative splice event junctions, (ii) create a separate data set of FESTs for each AS event type **(A)** Alternative donor, **(B)** Alternative acceptor, **(C)** Exon skipping and **(D)** Intron retention, (iii) Use BLAST to align FESTs to their corresponding event data sets, and (iv) AS events between two genes are classified as conserved, if each gene’s FESTs exhibit same AS type and also their FESTs match.

For each alternative splicing event, 30–300 bp of sequence from upstream and downstream exons, immediately flanking an intron defining the alternative junctions, were extracted (represented by black arrow in Figure 2). These flanking sequences that define splice junctions are termed flanking exon sequence tags (FESTs). Thus, each AS event is represented by a pair of FESTs and each FEST is tagged by its species name, gene name, coordinates of AS event (intron-exon junction positions of AS junctions), and FEST number (i.e., downstream FEST was numbered as 1 and upstream FEST as 2). FESTs from all species were grouped in to four separate databases, one for each AS event type (exon-skip, intron retention, alternative donor, and alternative acceptor). Each FEST in a database was searched against all other FESTs of same database with WU-BLASTN and WU-TBLASTX v2.0 (Gish, 1996). An AS event between two genes is considered as conserved, when these genes belong to same orthogroup and pair of FESTs of one gene align well with pair of FESTs of another gene (Figure 2). This analysis culminates in the identification of AS event clusters. Each cluster contains a set of conserved AS events represented within orthologs in up to nine species. It should be noted that the conserved AS events within a cluster may also include more than one paralogs gene from a single species. Thus, in the hypothetical cluster representing a conserved AS event between orthologous genes of only two species – for example, soybean and common bean, the conserved event may be represented within three member gene loci: a single common bean loci, and the two soybean homologs that had resulted from the soybean lineage specific WGD.

### Retrieving gene identifiers for MADS-box and SR gene families

Gene names for three type I (belonging to *Amborella trichopoda* and *Arabidopsis thaliana*) and 19 type II (belonging to *Amborella trichopoda*, *Arabidopsis thaliana*, *Populus trichocarpa*, and *O. sativa*) MADS-box gene sub-families classifications were retrieved from Amborella Genome Project (2013) (Supplementary Data 2). The gene identifiers belonging to these gene names for *Amborella trichopoda*, *Arabidopsis thaliana*, and *O. sativa* were retrieved from their original publications (Parenicova et al., 2003; Arora et al., 2007; Amborella Genome Project, 2013). Gene names listed in Amborella Genome Project (2013) for *P. trichocarpa* are gene identifiers obtained from an outdated version of the Richardson et al. (2011) (JGI 2.0.24), and were mapped to their corresponding identifiers in the JGI version 3 (JGI v3.0) of the *Populus* genome annotation used in this study. The identification mapping was performed by retrieving protein sequences for MADS-box genes from Ensembl release-24 (Kersey et al., 2014) and preforming BLASTP alignment of these sequences against protein sequences of JGI v3.0 genome annotation. Protein sequences and ID assignments were cross-verified by aligning the protein sequences to the *Arabidopsis* genome sequence and annotation resource at TAIR^2^.

Seven gene sub-families of Serine/Arginine-rich (SR) proteins belonging to *Arabidopsis thaliana*, *P. trichocarpa*, *G. max*, and *O. sativa* were taken from Richardson et al. (2011) (Supplementary Data 2). The gene identifiers used by Richardson et al. (2011) for *Arabidopsis thaliana*, *G. max*, and *O. sativa* were directly used in this study, while those for *P. trichocarpa* were obtained by mapping to the *Populus* genome annotation Version 3 (JGI v3.0), as described above for MADS-box genes.

### Building gene trees for MADS-box gene families

Multiple sequence alignments of full-length protein sequences were conducted for each sub-family with Muscle (Edgar, 2004a,b) with default parameters. Gene trees were constructed from these multiple sequence alignments for each sub-family with MEGA 6.0 (Tamura et al., 2013) using the maximum likelihood method with default parameters.

## Results

### Global transcriptome alignment and assembly

Transcriptome and genomic data were collected from nine angiosperm taxa constituting seven eudicots, one monocot (*O. sativa* – rice), and *Amborella trichopoda*, a pivotal species that is sister to all other angiosperms (Amborella Genome Project, 2013) and serves as an outgroup (Supplementary Table 1). The transcriptome collection includes sanger EST and mRNA sequence, 454, and Illumina RNA-seq from diverse tissue types (Supplementary Tables 2 and 3), which were rigorously quality-filtered, and assembled with a pipeline combining reference guided and *ab initio* assembly steps to fist create short-RNA-Seq read assemblies, followed by filtering and realignment with Program to Assemble Spliced Alignments (PASA) (Haas et al., 2003) alignments to identify and define species specific genome wide AS transcript isoforms (see Materials and Methods; Figure 1). PASA aligned assemblies were filtered to ensure that only isoforms with adequate read support for junctions (or retained introns) were retained, and all isoforms map to loci defining annotated protein coding genes (see Materials and Methods; Figure 1). For downstream AS analysis, only multi-exonic protein-coding genes with support from PASA transcripts were considered and these genes are referred to as expressed multi-exonic protein-coding genes (Supplementary Table 1).

### Intron retention is the most frequent AS event

PASA also generates an AS classification report. The PASA AS classification output was re-processed using a custom software pipeline to obtain AS events (Supplementary Figure 1; Supplementary Data 3) as defined in Wang and Brendel (2006). The four types of AS events examined in this study are: alternative donor site (AltD), alternative acceptor site (AltA), exon skipping (ExonS), and intron retention (IntronR). As illustrated in Table 1 and Supplementary Figure 2, IntronR is the most prevalent AS type among the seven species of eudicots, with *Arabidopsis* having the most abundant IntronR event category (65.3%). On average, more than half of the AS events are IntronR (56%), followed by AltA (21%), and AltD (14%), with ExonS (9%) being least frequent. These AS event frequencies are consistent with previous studies in plants (Wang and Brendel, 2006; Wang et al., 2008; Marquez et al., 2012).

**Table 1:** **Global AS events in nine angiosperms**.

AS type		Amborella	Arabidopsis	Soybean	Medicago	Rice	Common bean	Poplar	Tomato	Grape
AltA	Events (%)	9,427 (18.5)	5,377 (19.2)	13,056 (23.3)	6,115 (22.3)	6,110 (21.7)	5,675 (25.8)	7,807 (20.5)	3,918 (23.8)	7,373 (16.1)
	Genes (%)	5,342 (36.5)	3,929 (20.0)	8,774 (23.8)	4,256 (19.4)	4,212 (20.3)	3,982 (20.0)	5,383 (21.8)	3,069 (16.0)	4,794 (26.6)
AltD	Events (%)	7,166 (14.0)	3,168 (11.3)	9,055 (16.2)	4,237 (15.4)	3,514 (12.4)	3,575 (16.2)	4,541 (12)	2,127 (12.9)	5,315 (11.6)
	Genes (%)	5,342 (30.0)	3,929 (12.4)	8,774 (17.2)	4,256 (14.1)	4,212 (12.7)	3,982 (13.6)	5,383 (14.2)	3,069 (9.1)	4,794 (20.4)
ExonS	Events (%)	6,119 (12)	1,186 (4.2)	5,647 (10.1)	2,409 (8.8)	2,493 (8.8)	2,316 (10.5)	2,491 (6.6)	1,734 (10.6)	3,850 (8.4)
	Genes (%)	3,339 (22.8)	866 (4.4)	3,607 (9.8)	1,644 (7.5)	1,679 (8.1)	1,536 (7.7)	1,820 (7.4)	1,306 (6.8)	2,387 (13.2)
IntronR	Events (%)	28,328 (55.5)	18,325 (65.3)	28,262 (50.4)	14,710 (53.6)	16,100 (57.1)	10,440 (47.4)	23,190 (61)	8,655 (52.7)	29,184 (63.8)
	Genes (%)	8,693 (59.4)	8,438 (43.0)	12,870 (35.0)	7,047 (32.2)	7,177 (34.6)	5,798 (29.1)	10,412 (42.1)	4,888 (25.5)	10,071 (55.8)
Total	Events	51,041	28,057	56,021	27,472	28,218	22,007	38,030	16,435	45,723
	Genes (%)	10,292 (70.4)	10,398 (52.9)	18,476 (50.2)	9,781 (44.7)	9,641 (46.4)	8,932 (44.9)	13,152 (53.2)	7,503 (39.1)	11,628 (64.4)

### Up to 70% of expressed multi-exonic genes exhibit AS

Among all nine taxa, the fraction of multi-exon genes with at least one AS event is the highest in *Amborella* (70.4%), followed by *Vitis vinifera* – grape (64.4%), *P. trichocarpa* – popla*r* (53.2%), *Arabidopsis thaliana* (52.9%), *G. max* – soybean (50.2%), Oryza sativa – rice (46.4%), *Phaseolus vulgaris* – common bean (44.9%), *Medicago truncatula* (44.7%), and *Solanum lycopersicum* – tomato (39.1%) (Table 1 and Supplementary Figure 3). These percentages are conservative estimates because our analysis is restricted to only four AS event types (AltA, AltD, ExonS, and IntronR). A previous comprehensive AS study in *Arabidopsis* reported that 61.2% of expressed multi-exonic genes exhibit AS; however, the *Arabidopsis* study considered the top 10 most frequent types of AS to estimate AS frequency (Marquez et al., 2012).

### High-throughput pipeline for identifying conserved AS events

**Figure 3:**
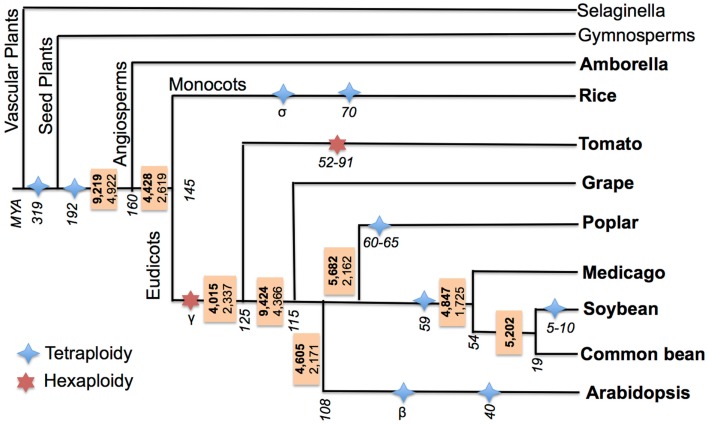
**Ancestral conserved AS events in flowering plants**. Species of interest are marked in bold. Ancestral AS events are reported in a box adjacent to each divergence node (events conserved with at least one other species other than the outgroup are in bold; events conserved within at least two other species are in plain font). Branch lengths are not proportional to length. Divergence of clades and WGD event timings are in MYA and are italicized, these are based on the following (Tuskan et al., 2006; Fawcett et al., 2009; McClean et al., 2010; Jiao et al., 2011; Woodhouse et al., 2011; Young et al., 2011; Paterson et al., 2012; Roulin et al., 2012; Amborella Genome Project, 2013).

Previous studies of cross-species AS event conservation primarily focused on two features: “conserved position” and “conserved junction” (Wang and Brendel, 2006; Wang et al., 2008; Darracq and Adams, 2013). In “conserved position” AS events, the same types of events should be present at the same position (precise splice-junction) between orthologous/paralogous genes, while “conserved junction” AS events are a relaxed version of conservation where the same type of events should be present at orthologous intron-exon junctions. Identifying “conserved position” AS events requires cross-species transcriptome alignments (Wang et al., 2008; Darracq and Adams, 2013) to determining the precise splice-junction positions between orthologous/paralogous genes. However, a “conserved position” approach is not ideal when comparing sequences between species separated by large evolutionary distances due to increased sequence divergence, which degrades at the sequence and intron-exon boundary level and complicates cross-species EST/transcript alignments. Thus, a “conserved junction” approach was chosen to identify conserved AS events across the large evolutionary distances separating the species examined in this study. Sequence conservation criteria for identifying conserved junctions are relaxed relative to those needed to define conserved positions, but require that the same type of events be present at orthologous intron-exon junctions. A high-throughput software pipeline for identifying conserved junction AS events was developed and tested by first identifying conserved AS events between soybean and common bean, and then implementing it to identify conserved AS across nine angiosperms species that define a broad evolutionary distance. Additionally, all of these species have undergone WGD events, some events are ancient and common to all species studied, while others are restricted to a single, or a few specific lineages (see Materials and Methods; Figure 3).

### More than 5,000 conserved AS event clusters between common bean and soybean

A total of 22,006 and 56,020 AS events across 8,931 and 18,475 unique loci were defined in common bean and soybean, respectively (Table 2 and Supplementary Data 4). Our pipeline revealed 5,202 conserved AS event clusters originating from 4,020 common bean and 5,671 soybean gene loci (Table 2), corresponding to 45 and 31% of multi-exon expressed genes with at least one AS event, respectively. IntronR was the most abundant conserved AS event type, followed by AltA, AltD, and ExonS, which is in alignment with overall proportion of AS events. To the best of our knowledge, this is the largest number of conserved AS events reported to date between two plant species, far exceeding results reported recently that describe 694 conserved AS events in 597 genes conserved *Arabidopsis thaliana* and *Brassica* (Darracq and Adams, 2013).

**Table 2:** **Conserved AS events between common bean and soybean at gene family level**.

		Common bean	Soybean
AltA	Conserved event clusters	1,563	1,563
	Conserved events (%)^a^	1,976 (35)	2,737 (21)
	Conserved event genes (%)^a^	1,518 (38)	2,123 (24)
	Total events	5,675	13,056
	Total genes	3,982	8,774
AltD	Conserved event clusters	807	807
	Conserved events (%)	1,023 (29)	1,434 (16)
	Conserved event genes (%)	809 (30)	1,111 (18)
	Total events	3,575	9,055
	Total genes	2,705	6,319
ExonS	Conserved event clusters	295	295
	Conserved events (%)	417 (18)	605 (11)
	Conserved event genes (%)	300 (20)	420 (12)
	Total events	2,316	5,647
	Total genes	1,536	3,607
IntronR	Conserved event clusters	2,537	2,537
	Conserved events (%)	3,798 (36)	5,286 (19)
	Conserved event genes (%)	2,381 (41)	3,255 (25)
	Total events	10,440	28,262
	Total genes	5,798	12,870
Total	Conserved event clusters	5,202	5,202
	Conserved events (%)	7,214 (33)	10,062 (18)
	Conserved event genes (%)	4,020 (45)	5,671 (31)
	Total events	22,006	56,020
	Total genes	8,931	18,475

*^a^Percentage is relative to total events and genes in each AS type*.

### Extensive species-specific AS events in WGD orthologs

Conserved AS events between a single gene in common bean and its orthologs resulting from a recent WGD in soybean were examined in 8,325 gene sets selected from the original 14,759 orthologous gene sets with 1 common bean gene: 2 soybean gene ortholog ratios. The original 14,759 gene sets were identified based on synteny gene analysis between common bean and soybean chromosomes (Schmutz et al., 2014). Out of these, 8,325 gene sets were chosen because they represent that subset of clusters where evidence exists for expression of all members of a gene set, at least one gene member is multi-exonic and there is evidence for a minimum of one AS event. Identified AS events within each group were used to classify the group into 1 of 5 categories based on their conservation status between orthologous gene copies of common bean and soybean. These categories are 1:2, 1:1, 0:2, 1:0, and 0:1, which represent the ratio of common bean gene members with a conserved AS event vs. the number soybean members of the gene cluster with conserved AS events. For example, placement of a gene cluster into the 1:2 category indicates that an AS event identified at a common bean gene locus has been conserved in both paralogs soybean loci that make up this gene cluster. There are 1,432 conserved AS events which are of category “1:2” in Table 3. The simplest interpretation of this result is that the AS event was present in the gene ortholog present in the most recent common ancestor (MRCA) to common bean and soybean, and that this AS event was maintained after speciation, and further retained in both paralogs resulting from the soybean WGD. Likewise, for category “1:1” there are 2,230 AS events representing conserved AS events between one gene copy of common bean and at least one of the two orthologous copies resulting from the soybean WGD in (category “1:1” in Table 3). There are 2,302 instances of AS events that are present in both parologs of a soybean gene but are absent in common bean (category “0:2” in Table 3). Two of the largest AS event categories are “1:0” (8,497 AS events only in common bean) and “0:1” (21,816 AS events only in soybean), suggesting rapid gain/loss of AS events among orthologous gene copies of common bean and soybean and also within gene pairs arising from WGD in soybean.

**Table 3:** **Conserved AS events in WGD orthologs**.

Gene copies in common bean: Gene copies in soybean													
	
		1:2		1:1		0:2		1:0		0:1		Total conserved	
		CB	SB	CB	SB	CB	SB	CB	SB	CB	SB	CB	SB
AltA	Conserved event clusters	489	489	581	581	0	540	NA	NA	NA	NA	1,070	1,070
	Events	599	1,218	653	693	0	1,288	1,938	0	0	4,878	1,252	1,911
	Genes	467	934	552	568	0	1,038	1,525	0	0	3,681	977	1,460
AltD	Conserved event clusters	252	252	354	354	0	332	NA	NA	NA	NA	935	935
	Events	320	660	422	416	0	797	1,431	0	0	3,799	1,075	1,109
	Genes	249	498	342	347	0	651	1,159	0	0	2,865	576	829
ExonS	Conserved event clusters	93	93	145	145	0	144	NA	NA	NA	NA	238	238
	Events	123	264	197	191	0	387	930	0	0	2,303	320	455
	Genes	92	184	136	139	0	272	662	0	0	1,577	223	317
IntronR	Conserved event clusters	598	598	1,150	1,150	0	1,286	NA	NA	NA	NA	1,748	1,748
	Events	865	1,772	1,454	1,458	0	3,236	4,198	0	0	10,836	2,319	3,230
	Genes	523	1,046	1,016	1,049	0	2,132	2,773	0	0	5,961	1,433	1,984
Total	Conserved event clusters	1,432	1,432	2,230	2,230	0	2,302	NA	NA	NA	NA	3,662	3,662
	Events (%)	1,907 (15)	3,914 (11)	2,726 (21)	2,758 (8)	0	5,708 (17)	8,497 (65)	0	0	21,816 (64)	4,633 (35)	6,672 (20)
	Genes (%)	1,132	2,264	1,761	1,883	0	3,464	4,274	0	0	9,053	2,542 (31)	3,757 (23)

### More than 27,000 conserved AS event clusters among nine angiosperm species

Conserved AS events among nine angiosperm species were identified using our pipeline and are classified into conserved AS event clusters (Supplementary Data 4). Each conserved AS event cluster has AS events that are conserved between genes belonging to the same orthogroup (see Materials and Methods), although these gene clusters may include one or more paralogs genes from a single species. At any rate, each cluster represents conservation of a single AS event impacting a single junction (or junction pair) within the cluster. Therefore, a 3-member cluster represented by an AS event at a common bean gene loci that is conserved in both of two post-WGD soybean homeolgs genes represents a single AS event. There are 27,120 conserved AS event clusters between at least two of the nine angiosperm taxa used in this study (Table 4). As expected, the number of events conserved between species is inversely proportional to the number of species assayed, with the most (16,416; 60.5%) conserved events identified between only two species and only a modest number (101; 0.4%) conserved across all nine species (Table 4). Of these, IntronR is the most common AS event (65.6%) followed by AltA (20.5%), AltD (10.0%), and ExonS (3.9%) of all events (Table 4). The proportion of expressed protein-coding multi-exonic genes with at least one conserved AS in at least one other species is highest for grape (36.2%), followed by *Amborella* (34.1%), poplar (29.4%), common bean (27.7%), soybean (26.7%), *Arabidopsis* (24%), *Medicago* (23.7%), tomato (17%), and rice (16.7%) (Table 5).

**Table 4:** **Conserved AS events across angiosperms at gene family level**.

Number of species	AltA	AltD	ExonS	IntronR	Total	Total (%)
2	3,691	1,945	808	9,972	16,416	60.5
3	1,053	435	142	4,246	5,876	21.7
4	362	141	36	1,855	2,394	8.8
5	217	70	22	859	1,168	4.3
6	116	57	13	413	599	2.2
7	64	34	13	254	365	1.3
8	39	24	10	128	201	0.7
9	20	8	5	68	101	0.4
Total	5,562	2,714	1,049	17,795	27,120	
Total (%)	20.5	10.0	3.9	65.6	20.5	

**Table 5:** **Genes with conserved AS events across at least one other species**.

AS type	Number of genes with conserved AS events									
	Total	Amborella	Arabidopsis	Soybean	Medicago	Rice	Common bean	Poplar	Tomato	Grape
AltA	14,745	1,322	1,030	3,563	1,690	830	2,040	1,848	893	1,529
AltD	7,323	644	458	1,882	904	360	1,099	858	365	753
ExonS	2,796	305	120	689	310	136	433	312	176	315
IntronR	39,992	4,326	4,009	6,945	3,864	2,896	3,648	6,051	2,434	5,819
Total	50,792	4,993	4,710	9,827	5,182	3,511	5,517	7,256	3,258	6,538
Total (%)^a^		34.1	24.0	26.7	23.7	16.9	27.7	29.4	17.0	36.2

*^a^Percentage is based on expressed protein coding multi-exonic genes*.

The percentage of conserved AS events relative to the total number of conserved AS events was calculated for each pair-wise species comparison (Supplementary Figure 4). Of the conserved AS events identified in common bean that are conserved in one or more additional species, the largest fraction of these (68%) is conserved with soybean. This also accounts for the single pair-wise comparison among all nine taxa that has the highest level of conservation. The pair-wise comparison identifying the second highest fraction of conserved events occurs between *Medicago* and soybean, with 58% of the conserved AS events within *Medicago* conserved with soybean (Supplementary Figure 4). This is not unexpected owing in part to their close phylogenetic relationship (Figure 3) and also the availability of deep transcriptome data (Supplementary Table 3). Interestingly, the majority of the species examined share their largest fraction of conserved AS events with grape (Supplementary Figure 4). One possible explanation for this is that grape has a superior transcriptome collection compared to all other species – 114.7M 100 bp paired-end RNA-seq reads (23 GB) generated by pooling RNA from 45 samples representing various developmental stages as detailed in Supplementary Table 8 (Venturini et al., 2013). Other possible explanations would be that AS fractionation in grape may be very low compared to other species (Freeling, 2009). Of all angiosperms in this study (Figure 3), *Amborella* is the only species that has not undergone any lineage-specific WGD events in addition to the ancient WGD shared by all angiosperms (Amborella Genome Project, 2013), while grape has undergone only one whole genome triplication (i.e., two WGDs in close succession) (Jiao et al., 2012), and the rest of the species have undergone at least two or three WGD events (Figure 3), which may have lead to high AS fractionation in these species relative to grape.

### Ancestral angiosperm AS events

Ancestral AS events were estimated at each of the nodes within the species tree of the nine angiosperm species included in this study (Figure 3). The ancestral AS event numbers reported in boxes at each node were calculated by requiring that each AS event be conserved between an outgroup species and at least one other ingroup species. There are 9,219 AS ortholog event clusters identifying unique 9,219 AS events conserved between *Amborella* and at least one orthologous loci from one or more other species in the study, indicating these events may have been present in the MRCA of angiosperms. The highest number of conserved AS events (9,424) is seen at the node between grape and eurosids. This extent of conservation may reflect the comprehensive nature of the grape transcriptome sequence collection, or may suggest that the grape genome is evolving slowly and has maintained much of the AS events in common with its MRCA with other eurosids.

All other nodes have fewer than 6,000 AS events. Because the rate of convergent evolution of AS events in plants is not known, too what extent all of these events are strictly ancestral vs. what fraction actually represents convergent gains of AS remains unclear. To minimize mistaking convergently evolved AS events for the ancestral state of the MRCA for each node, we determined the number of AS events that were simultaneously conserved between an outgroup species and at least two other ingroup species. Using this criterion, 4,922 events are conserved between *Amborella* and two or more angiosperms in this study, thus reducing the number of conserved events by about half compared to our previous estimate. This analysis thus provides a lower bound for the number of AS events (4,922) that existed within the MRCA of *amborella* and all sister angiosperm species. The number of conserved events at each of the other nodes was similarly reduced when using the more stringent classification criterion (Figure 3), and further illustrates the dynamic gains and losses of AS events across species.

### Alternative splicing in sub-families of MADS-box genes

MADS-box genes are sub-divided into two types – type I and type II. The extent of AS in MADS-box genes and their cross-species conservation is studied in three type I and 19 type II sub-gene families as classified by the Amborella Genome Project (2013) (Supplementary Data 2).

The MADS-box type I gene family in *Amborella* and *Arabidopsis* is represented by 12 and 57 gene members, respectively. Only four (33.3%) of the *Amborella* and 13 (22.8%) of the *Arabidopsis* MADS-box type I genes are shown to be multi-exonic and expressed based on the transcriptome data we used (Supplementary Table 9). These low gene numbers are in agreement with previous study that reported low exon count (mostly one or two) among genes of MADS-box type I (Nam et al., 2004; Gramzow and Theissen, 2010). Among expressed multi-exonic genes, 4 (100%) from *Amborella* and 4 (30.8%) from *Arabidopsis* show evidence for at least one AS event (Supplementary Table 9). Among the three sub-families (α, β, γ) of MADS-box type I genes, only *Arabidopsis* provides evidence for AS in the α sub-family, only *Amborella* provides evidence for AS in the β sub-family, while both *Arabidopsis* and *Amborella* genes from the γ sub-family exhibit AS (Supplementary Figure 11 and Supplementary Table 9). Although at least one gene from the γ sub-family in both *Arabidopsis* and *Amborella* undergoes AS, there was no evidence of cross-species AS event conservation.

Unlike MADS-box type I genes, larger numbers of MADS-box type II genes are multi-exon and shown to be expressed in the RNA-Seq data used for our analysis: 87% (20), 100% (45), 97.3% (36), and 88.6% (39) in *Amborella*, *Arabidopsis*, rice, and poplar respectively (Supplementary Table 10). The percent of expressed multi-exonic genes with at least one AS event is highest for *Amborella* (100%), followed by *Arabidopsis* (77.1%), rice (66.7%), and poplar (56.4%) (Supplementary Table 10). There are five AS events – four intron retention and one alternative acceptor – conserved between at least two species in MADS-box type II sub-families, AG, ANR1, AP1, and STK (Supplementary Data 5, Supplementary Figure 12, and Supplementary Table 10). Of these conserved AS events, two AS events in ANR1 (intron retention) and STK (alternative acceptor) sub-families are presented in *Amborella* and in at least one other angiosperms (either *Arabidopsis* or rice or poplar), indicating these events may have been present in the MRCA of angiosperms.

### High alternative splicing conservation among SR genes

At least 89% of expressed multi-exonic SR genes show evidence for alternative splicing, with the highest in *soybean* (95.7%), followed by rice (95.5%), poplar (89.5%), and *Arabidopsis* (88.9%) (Supplementary Table 11). These high rates of AS in SR genes are in alignment with previous studies (Richardson et al., 2011). Along with high rates of AS, SR genes also have high rates of cross-species AS conservation. Out of seven SR gene sub-families in this study (Supplementary Data 2), six of them have at least one AS event conserved in more than one species. Overall, there are 34 AS events (Supplementary Data 6) that are conserved in at least two species, with 11 AS events being conserved in all four species. There are 19 AS events (Supplementary Data 6) that are conserved between a monocot (rice) and at least one other eudicot (*Arabidopsis*, poplar, and soybean), suggesting these events may have been present in the MRCA of monocots and eudicots.

## Discussion

### Frequency of genes with AS

This manuscript reports a computational identification of AS isoforms within the genes of nine diverse angiosperms using gene annotations were supported by NGS data. In addition, we describe a comparative genomics analysis that identifies conserved AS events between two or more members of all nine species studied. To our knowledge, this study reports the largest AS discovery and conserved event analysis conducted in plants. The proportion of multi-exonic genes exhibiting AS within *Amborella* and grape are 70.4 and 64.4%, respectively, both of which exceeds the current estimate in *Arabidopsis* (61.2%) (Marquez et al., 2012). However, because this analysis considered only the four most frequent types of AS events (exon-skip, intron retention, alternative donor, alternative acceptor) of the possible (Table 1 and Supplementary Figure 1), these results are still likely to underestimate the extent of AS in plant transcriptomes. We observe a range in the proportion of expressed multi-exonic genes exhibiting AS within the nine angiosperm taxa (40–70%), which likely reflects differences in the individual transcriptome resources available at the time this analysis was conducted. Long paired-end reads improve mapping and facilitates AS event detection, while the diverse tissue sampling allows identification of events that may be restricted to specific tissues. In contrast to *Amborella* and grape, the majority of the tomato RNA-seq data is represented in 50 bp reads that were sampled from fruit tissue, which limits both transcript assembly and diversity.

Previous studies investigating genome-wide conserved AS events in plants are limited to at most three species (Baek et al., 2008; Darracq and Adams, 2013), and relied on cross-species transcriptome alignments (Wang et al., 2008; Darracq and Adams, 2013), or pair-wise comparisons of genes to identify orthologs followed by examination for conserved AS events (Wang and Brendel, 2006). Darracq and Adams (2013) identified AS events conserved between *Arabidopsis thaliana* and *Brassica* through alignments of these species transcriptome sequences to *Arabidopsis* gene models. Same-species alignments (i.e., *Arabidopsis* ESTs aligned to *Arabidopsis* genes) mapped ~70% of the sequences at high-stringency, while only ~40% of *Brassica* sequences could be aligned with low stringency alignment parameters – a 30% reduction alignments between species. The failure to align many of the *Brassica* sequences likely reflects sequence divergence relative to Arabidopsis since ~93% of gene families are common between *Arabidopsis* and *Brassica rapa* (Wang et al., 2011); thus, this methodology may be failing to identify conserved AS events. Additionally, performing pair-wise comparisons of close homologs is not easily scalable to simultaneously assessing a large number of taxa because plants often have lineage-specific WGD events, and shared ancient WGD events that can confound the assignment of gene pairs (Gabaldón and Koonin, 2013). This can be particularly problematic for high-throughput sequence search strategies such as the reciprocal BLAST based pair-wise comparisons used by Wang and Brendel (2006). This is also an inefficient strategy for large multi-species datasets since the number of pair-wise comparisons required grows exponentially with the number of species. The strategy to identify conserved AS events presented in this study first identifies AS events within each species by aligning same species transcriptome and genome data, and then compares AS events associated with orthologous gene collections. Our strategy for identifying conserved AS events does not rely on either cross-species alignments or pair-wise gene comparisons. Rather, transcriptome to genome alignments identify AS splicing events for each species and these are used to construct a FESTs dataset for each event type for each species examined (see Materials and Methods; Figure 2). FESTs AS event datasets were compared using TBLASTX and BLASTN to identify all possible events conserved between two or more species. TBLASTX alignments allow detection of alignments between orthologous sequences that have high sequence divergence at the nucleotide level but are conserved at the amino acid level. Conserved events define AS event clusters, which reconciled with OrthoMCL gene clusters to identify AS events that truly represent identical events between othologous genes.

### Identification of conserved AS events between common bean and soybean

This study reports 5,202 conserved AS event clusters between common bean and soybean, which is the largest number of conserved AS events between two plant species reported to date. This number is much higher than the 694 reported conserved events between *Arabidopsis thaliana* and *Brassica* species (Darracq and Adams, 2013). The estimated times of divergence between common bean and soybean and between *Arabidopsis thaliana* and *Brassica* species, is 19 MYA (Lavin et al., 2005) and 20 MYA (Yang et al., 1999), respectively. These estimates are similar, suggesting that differences in divergence times are not likely to be a major contributing factor to the disparity in the number of conserved AS events identified between common bean and soybean compared to those identified between *Arabidopsis thaliana* and *Brassica* (Darracq and Adams, 2013). As previously mentioned, the high numbers of conserved AS events identified compared to that of Darracq and Adams (2013) may reflect differences in the transcriptome resources and the AS identification strategies used in each study. Darracq and Adams (2013) examined only ESTs, whereas our study includes ESTs, mRNA, and high-depth RNA-seq data from diverse tissues. Additionally, specific features of our conserved AS identification algorithms discussed above also affect the identification of conserved AS events. Despite having collections of RNA-seq data from similar tissue types from both common bean and soybean (Supplementary Table 3), only 33 and 11% of AS events, respectively, are conserved (Table 2), suggesting that each species harbors substantial numbers of lineage-specific AS events.

### Identification of conserved AS events in WGD orthologs

Common bean and soybean provide a model system to examine conserved AS between two closely related species where the genes in one species (common bean) have orthologous relationships to gene pairs that resulted from a WGD event (soybean). Conserved AS events were investigated within 8,325 orthologous gene sets composed of a common bean gene and its two homeologous orthologs in soybean. Only 36 and 19% of AS events in common bean and soybean, respectively, are conserved between a common bean gene and at least one member of the homeologous gene pair representing the orthologous soybean gene pair (1:2 and 1:1 categories of Table 3). These conserved AS event ratio categories represent the AS events that were likely present in the MRCA of common bean and soybean. Thus, the observation that ~65% of AS events (1:0 and 0:1 categories of Table 3) are associated solely with common bean or soybean, respectively, suggests that there were rapid AS gains/losses within these species after their divergence from a MCRA, and some of this may reflect fractionation after the soybean WGD.

Approximately, 17% (5,708) of the conserved AS events are absent in common bean but present in both homeologous gene copies of soybean (0:2 category; Table 3). There are four possible scenarios that could account for this: (i) AS events may have independently arisen in duplicate copies at the same position in soybean, (ii) AS events would have been present in the MRCA of common bean and soybean but were lost in common bean after its divergence from the soybean lineage, (iii) AS events are not present in the MRCA of common bean and soybean but were formed after their divergence within the soybean lineage but prior to the soybean-specific WGD event, such that both homeologues have the event, and (iv) the AS event is actually conserved in common bean but was not recovered in our transcriptome dataset. Only the second of these four possible explanations is testable with our data and analysis. If an AS event was present in MCRA of soybean and common bean, but lost in common bean subsequent to its divergence from soybean, then it’s possible that this event will be conserved within a close outgroup. Analysis of this type identified that approximately 43% (2,460) of these events (Supplementary Table 5) are conserved within at least one other angiosperm examined in this study, suggesting that loss of these events from common bean after it’s divergence from soybean is the most parsimonious explanation for it’s absence. However, the remaining three explanations could account for this observation and none can be discounted at this point. Indeed, by the same argument, the remaining events (57%) might well have arisen from any of these three scenarios, further underscoring that they may be active in the evolution of alternate splice isoforms.

### Identification of conserved AS events among nine diverse angiosperm taxa

Our analysis has identified 27,120 AS events (Table 4) found to be conserved between at least two of nine angiosperm taxa using the conserved AS event identification pipeline. This is the largest number of conserved AS events reported to date, a ~38X increase relative to conserved AS events previously identified (Darracq and Adams, 2013). Additionally, this is the first study to investigate genome-wide conserved AS events in plants across more than three species, and includes *Amborella*, a plant species sister to all extant angiosperms as an outgroup. There are 101 AS events that are conserved across all nine angiosperms including species at long evolutionary distances. Furthermore, this is the first study to reconstruct the ancestral state of AS events (9,219) in the MRCA of angiosperms (using *Amborella* as an outgroup) and also reports the largest number of AS events (4,015) conserved between a monocot (rice) and at least one eudicot (Severing et al., 2009). GO category enrichment analysis (for details, see Supplementary Methods 1.2) was used to investigate whether or not specific GO categories are overrepresented among the 1,099 *Arabidopsis* genes found to share conserved AS events with six or more angiosperms examined in this study. Several general terms describing biological processes, cellular component, and molecular function were apparently enriched among this set of genes (Supplementary Table 6 and Supplementary Figures 5–7), suggesting that common biological processes may be influencing, in part, conservation of specific AS isoforms. Interestingly, examining GO enrichment in 2,264 soybean genes where conserved AS events are present in two WGD paralogs of soybean and their corresponding ortholog in common bean (1:2 category; Table 3) identified many of the same GO categories as overrepresented (Supplementary Table 7 and Supplementary Figures 8–10), which suggests that genes with AS events that tend to be conserved across species are also preferentially being retained in gene copies derived from WGD. This approach provides new insight into AS conservation in plants across large phylogenetic distances, and across multiple lineages. Because our methodology is amenable to inclusion of all sequence platform data types and is efficient, it maximizes the use of available data and increases the identification of orthologous relationships and AS event discovery. This high-throughput AS conservation methodology is easily scalable to any future analyses involving a greater number of species representing a wide phylogenetic distribution, and is certainly not restricted to the plant kingdom.

### Application of the identification of conserved AS events

Our search for conserved AS events across nine plant species that represent a large phylogenetic distance has revealed several thousand AS events conserved among nine plant species, which implies that these events are important and have been retained during the course of evolution. Previously, comparative AS studies have helped to identify important events and prioritize them for further characterization. For example, Fu et al. (2009) compared an exon-skipping event in *TFIIIA* of *Arabidopsis thaliana* with other species, including monocots, eudicots, mosses, and early vascular plants, and found this event to be highly conserved. This evidence prompted further investigation that revealed a novel exonization of 5S-RNA that provides the basis for post-transcriptional regulation of TFIIIA, which is a transcription factor required for 5S-RNA transcription (Fu et al., 2009). Our study also identified this same *TFIIIA* exon-skipping event, confirming that our pipeline is efficiently identifying bonafide cross-species AS events. Molecular characterization studies similar to Fu et al. (2009) could be initiated on thousands of the conserved AS events that have been identified during this analysis.

Identifying conserved AS events can identify gene families where AS events are common among its members and enables examination of AS conservation rates in these gene families both within and across species. Additionally, one can investigate correlations between the number of genes exhibiting AS vs. gene family size. Evidence from previous studies suggests that some gene families show higher rates of AS compared to others (Richardson et al., 2011). One such gene family is the Serine/Arginine-Rich protein gene family (SR proteins) in plants. SR proteins function in spliceosome assembly, as well as constitutive and alternative splicing of pre-mRNAs, including their own transcripts (Richardson et al., 2011). Compared to vertebrates, angiosperms have nearly twice the number of genes encoding SR proteins, and AS within SR protein-encoding genes is common. For example, *Homo sapiens* have 11 SR genes, while *Arabidopsis thaliana* and *O. sativa* have 18 and 22 SR genes, respectively (Richardson et al., 2011); and 16 of 18 *Arabidopsis* SR protein genes undergo AS (Richardson et al., 2011). Using our conserved AS event identification pipeline, one can identify gene families that, similar to the family of SR proteins, undergo widespread AS and further investigate these events for functional relevance. Our study identified 11 of 18 SR proteins that have conserved AS events with at least one other species, with the majority of them exhibiting conservation in at least six other angiosperms.

Ancestral reconstruction of gene family content and examination of gains and losses of genes relative to the MRCA of various plant lineages gives interesting insights into how these changes may have been involved in the evolution of new traits, especially key innovations. To accurately draw conclusions about gene gains and losses, each species should have nearly complete gene sets, and these are increasingly available with improvements in sequencing technology. Similarly, to accurately identify lineage-specific gains or losses of AS events and to investigate their implications, one needs to have similarly comprehensive and uniform transcriptome datasets. Currently, complete transcriptome datasets across multiple species are not publicly available. Although, we were able to estimate the ancestral state of AS events at various nodes using a parsimony argument (Figure 3), it is not possible for us to infer the exact origin of these because our transcriptome datasets are neither uniform nor comprehensive. It is important to continually investigate gains and losses of AS events across various lineages and their functional implications as transcriptome datasets increase in depth and sampling diversity. Our comparison of AS in WGD gene copies of soybean with common bean identified an overrepresentation of GO terms among soybean genes having conserved AS events between its WGD paralogous copies and its ortholog in common bean (Supplementary Table 7). It would be interesting to examine AS conservation and similar GO term enrichment within one or more groups of closely related non-legume species that have independent WGD histories. A comparison of these data will identify those genes most likely to retain, lose, or gain novel AS events after WGD or gene family expansion. Insight into the evolution of AS after gene/genome duplication will complement efforts in understanding the predictability of gene loss/retention during genome fractionation following a WGD event (Buggs et al., 2012).

## Conflict of Interest Statement

The authors declare that the research was conducted in the absence of any commercial or financial relationships that could be construed as a potential conflict of interest.

## Supplementary Material

The Supplementary Material for this article can be found online at http://journal.frontiersin.org/article/10.3389/fbioe.2015.00033

## Footnotes

^1^http://trinityrnaseq.github.io/#genome_guided

^2^http://www.arabidopsis.org/
