# Supplementary material for: Genome-Wide Identification of Evolutionarily Conserved Alternative Splicing Events in Flowering Plants
Source: Front Bioeng Biotechnol. 2015 Mar 26;3:33. doi: 10.3389/fbioe.2015.00033 (PMC4374538; doi:10.3389/fbioe.2015.00033)
Supplement: Supplementary file 1 [file Data_Sheet_1.ZIP › Chamala_etl_Supplementary_Material_V20.docx]

***Supplementary Material***

**Genome-wide identification of evolutionarily conserved alternative splicing events in flowering plants**

**Srikar Chamala^1^, Guanqiao Feng^2^, Carolina Chavarro^3^, W. Brad Barbazuk^1,4^***

^1^Department of Biology, University of Florida, Gainesville, FL, USA

**^2^**Graduate Program in Plant Molecular and Cellular Biology, University of Florida, Gainesville, Florida 32611

**^3^**Center for Applied Genetic Technologies, University of Georgia, Athens, GA 30602

^4^Genetics Institute, University of Florida, Gainesville, FL, USA

*** Correspondence:** W. Brad Barbazuk, Department of Biology, University of Florida, Gainesville, FL 32611, USA.

[bbarbazuk@ufl.edu](mailto:bbarbazuk@ufl.edu)

1. **Supplementary Methods**

## RNA-seq data processing and assembly

Three different methodologies were implemented for assembling RNA-seq data (Supplementary Table 1) to maximize the recovery of all possible isoforms. Before assembly, raw RNA-seq data were screened to remove adapter sequences using Cutadapt v1.1 (Martin, 2011) with the following parameters: error-rate=0.1, times=1, overlap=5, and minimum-length=0. Adapter trimmed sequences were quality trimmed with Trimmomatic v0.22 (Lohse et al., 2012) using the following parameters: HEADCROP:0, LEADING:3, TRAILING:3, SLIDINGWINDOW:4:15, and MINLEN:40.

## Calculating maximum intron size

Maximum intron sizes used as input to alignment programs were directly calculated for each genome. Briefly, the 99^th^ percentile for intron length for each genome was calculated from the available annotation. Added to this value was the mean exon size for the genome in question (Supplementary Table 4).

## Short-read assembly strategy

PASA (Program to Assemble Spliced Alignments) (Haas et al., 2003) is a utility that performs accurate splice-aware alignments between transcripts and genome reference sequences defining transcripts and AS isoforms, and further classifies AS events. The complexity of clustering and assembling overlapping alignments resulting from massive numbers of 454 and Illumina RNA-seq reads is beyond PASA. To overcome this, reads from 454 and Illumina were pre-assembled with both *de novo* and genome-guided transcriptome assembly methodologies, which effectively reduced to collection from hundreds of millions of short transcriptome reads to a few hundred thousand longer assemblies transcripts (Rhind et al., 2011; Haas et al., 2011). These transcript assemblies were combined with available sanger EST and mRNA sequences, and were aligned using the splice-aware aligners GMAP (Wu and Watanabe, 2005) and BLAT (Kent, 2002) to corresponding reference genomes, and these were finally assembled by PASA (Figure 2) (Rhind et al., 2011; Haas et al., 2011).

## Trinity genome-guided assembly

Only AS events associated with protein-coding genes were considered for this study. Gene sequences with 2000 bp of flanking regions were extracted from the appropriate genome assemblies. If two genes overlapped the whole region defined by the non-overlapping edges + 2000bp on either side of these was extracted. If addition of the 2000 bp flanking regions resulted in neighboring genes overlapping then the reduced to 1000bp.

The GSNAP v2012-07-12 (Wu and Nacu, 2010) alignment program was used to align RNA-seq data sets against the collection of extracted gene sequences and to the original genome where sequence corresponding to the extracted regions were hard masked. Including masked gene regions avoids false alignments to genic regions, i.e., the cases where an RNA-seq read would have top alignment to a non-genic region and if, in the absence of a genome sequence, it would align to genic region as the top hit. This approach compared to directly aligning RNA-seq reads to whole genome may help minimize merging of transcript assemblies from overlapping genes in the genome. GSNAP alignment parameters were: batch=5, suboptimal-levels=0, novelsplicing=1, local-splice-penalty=0, distant-splice-penalty=1, npaths=5, quiet-if-excessive, max-mismatches=0.05, nofails, format=sam, sam-multiple-primaries, split-output, orientation=FR, pairexpect=200, and pairdev=25; localsplicedist and pairmax-rna were both set to the 99^th^ percentile of maximum intron sizes (Supplementary Table 4). All alignments of RNA-seq datasets were de-duplicated and merged using the MarkDuplicates and MergeSamFiles utilities of the Picard software package v1.72 (<http://picard.sourceforge.net>). Genome-guided Trinity v20130225 (<http://trinityrnaseq.sourceforge.net/genome_guided_trinity.html>) was run on the merged and cleaned GSNAP alignments using default parameters.

## Trinity *de novo* assembly

All cleaned RNA-seq data sets for a given species were merged and Trinity's *in silico* read normalization (Haas et al., 2013) was run using the following parameters: JM 175G, max_cov 50, JELLY_CPU 20, min_kmer_cov 2, pairs_together, and PARALLEL_STATS. This normalization process reduces the number of reads, which substantially reduced the run-time and memory requirements for the Trinity assembly (Grabherr et al., 2011).

## Cufflinks assembly

Cleaned RNA-seq datasets were aligned to their corresponding reference genome sequences with Tophat v2.0.9 (Kim et al., 2013) using the following parameters: read-mismatches 5, read-gap-length 3, read-edit-dist 5, read-realign-edit-dist 0, mate-inner-dist 100, mate-std-dev 50, max-intron-length 8967, num-threads 20, max-multihits 5, library-type fr-unstranded, and GTF; max-segment-intron and max-coverage-intron were set to the 99^th^ percentile of intron sizes Supplementary Table 4. All read alignments were de-duplicated and merged using MarkDuplicates and MergeSamFiles utilities of the Picard software package v1.72 (<http://picard.sourceforge.net>) and assembled using Cufflinks v2.1.1(Trapnell et al., 2012b, 2012a) in a reference guided fashion: GTF-guide, frag-bias-correct, min-isoform-fraction 0.05, multi-read-correct, upper-quartile-norm, library-type fr-unstranded, min-frags-per-transfrag 10, min-intron-length 50, and no-faux-reads; max-intron-length for each species was set to the 99^th^ percentile of intron sizes Supplementary Table 4. Cufflinks transcript assemblies were kept if ‘full_read_support’ flag status was ‘yes’ and had FPKM of at least 0.1.

## GO category enrichment

Two GO category enrichment analyses were performed in this study. The first analysis examines genes in *Arabidopsis* that have conserved AS events across at least six angiosperms. The second analysis examines soybean genes that have at least one AS event conserved in two WGD paralogs of soybean and their corresponding ortholog in common bean.

Protein sequences for 27,416 and 54,175 genes in *Arabidopsis thaliana* and *Glycine max,* respectively, were extracted from the genome annotation versions described in Supplementary Table 1. In cases of genes with multiple isoforms, only the one predicted to produce the longest protein was retained. These protein sequences were aligned by NCBI-BLASTP (Altschul et al., 1990, 1997) to the NCBI's nr (non-redundant) database using the following parameters: -m 7 -v 20 -b 20, and results were output in xml format. The alignment results were imported into Blast2GO (Conesa et al., 2005). Using Blast2GO’s default parameters, annotations and GO terms were extracted followed by GO category enrichment analysis using its Fisher’s exact test module.

1. **Supplementary Data**

**Supplementary Data 1**. “Supplemental_Data_1_Nine_Angiosperms_Orthogroups.xlsx” – Gene orthogroups from OrthoMCL belonging to nine angiosperms used in this study.

**Supplementary Data 2**. “Supplemental_Data_4_MADS-box_SR_Gene_Families.xlsx” – Sub-families of MADS-box type I (for *Amborella trichopoda* and *Arabidopsis thaliana*), type II (for *Amborella trichopoda*, *Arabidopsis thaliana*, *Populus trichocarpa,* and *Oryza sativa*) and SR protein (for *Arabidopsis thaliana, Glycine max*, *Populus trichocarpa*, and *Oryza sativa*) genes as classified by Amborella Genome Project (2013) and Richardson et al. (2011).

**Supplementary Data 3**. “Supplemental_Data_3_Nine_Angiosperms_AS_Events.zip” – A tab delimited file containing AS events in genes belonging to all nine angiosperms used in this study.

**Supplementary Data 4**. “Supplemental_Data_4_Nine_Angiosperms_Conserved_AS_Events.xlsx” – Conserved AS event clusters among nine angiosperms used in this study.

**Supplementary Data 5**.

“Supplemental_Data_5_MADS_BOX_TYPEII_Conserved_AS_Events.xlsx” – Conserved AS event clusters among genes of MADS-box type II sub-families of *Amborella trichopoda*, *Arabidopsis thaliana*, *Oryza sativa*, and *Populus trichocarpa*.

**Supplementary Data 6**. “Supplemental_Data_6_SR_Proteins_Conserved_AS_Events.xlsx” – Conserved AS event clusters among genes of SR proteins sub-families of *Glycine max*, *Populus trichocarpa*, *Arabidopsis thaliana*, and *Oryza sativa*.

1. **Supplementary Figures and Tables**

## Suplementary Tables

**Supplementary Table 1. Genome sequence and annotation resources.**

| Species | Protein Coding Genes | | | Source of Collection |
| --- | --- | --- | --- | --- |
|  | All | Multi-  exonic | Expressed  Multi-exonic |  |
| *Amborella*  *trichopoda*  (Amborella) | 26,846 | 17,187 | 14,626 | <http://amborella.org/>  Annotation version 1.0  (Chamala et al., 2013; Amborella Genome Project, 2013) |
| *Arabidopsis*  *thaliana*  (Arabidopsis) | 27,206 | 21,236 | 19,637 | Phytozome 9.0  Annotation TAIR 10  (Swarbreck et al., 2008) |
| *Glycine*  *max*  (Soybean) | 54,175 | 45,369 | 36,789 | Phytozome 9.0  Annotation Version 1.1  (Schmutz et al., 2010) |
| *Medicago*  *truncatula*  (Medicago) | 50,895 | 39,323 | 21,889 | Mt4.0v1  <http://www.jcvi.org/medicago/>  (Young et al., 2011; Tang et al., 2014) |
| *Oryza*  *sativa*  (Rice) | 38,867 | 29,098 | 20,760 | Phytozome 9.0  MSU Release 7.0  (Excluding ChrUn and ChrSy molecules)  (Ouyang et al., 2007) |
| *Phaseolus*  *vulgaris*  (Common bean) | 27,198 | 22,620 | 19,910 | Phytozome 9.0  Annotation version 1.0  <http://www.phytozome.net/commonbean> |
| *Populus*  *trichocarpa*  (Poplar) | 41,336 | 33,412 | 24,712 | Phytozome 9.0  Annotation version JGI v3.0  - assembly v3  (Tuskan et al., 2006) |
| *Solanum*  *lycopersicum*  (Tomato) | 34,728 | 26,220 | 19,168 | Phytozome 9.0  Annotation version ITAG2.3  (Sato et al., 2012) |
| *Vitis*  *vinifera*  (Grape) | 26,347 | 24,448 | 18,053 | Phytozome 9.0  Annotation version as of March 2010  (Jaillon et al., 2007) |

**Supplementary Table 2. EST, mRNA, 454, and RNA-seq sequence data summary.**

| Species | EST | mRNA | 454 Sequence | RNA-Seq |
| --- | --- | --- | --- | --- |
| *Amborella trichopoda* | 38,147 | 14 | 2,243,371 | 2X72: 201.9M (30.2 Gb)  2X101: 242.7M (49Gb) |
| *Arabidopsis thaliana* | 1,529,700 | 81,157 | NA | 1X82bp: 89.1M (7.31 Gb)  1X101bp: 948.4 M (95.8 Gb)  2X76: 158.7M (24.1Gb) |
| *Glycine max* | 1,461,722 | 2,231 | NA | 2X101: 593M (119.8 Gb)  1X76: 58.4M (4.4 Gb) |
| *Medicago truncatula* | 269,501 | 46,682 | NA | 2X101: 79.1M (16Gb)  1X101: 489.8M (49 Gb) |
| *Oryza sativa* | 89,943 | 33,547 | NA | 2X104: 64.1M (13Gb)  2X75: 81M (12.3Gb) |
| *Phaseolus vulgaris* | 125,490 | 381 | NA | 2X100: 371M (74.2Gb) |
| *Populus trichocarpa* | 89,943 | 393 | NA | 2X101: 563.8 M (114 Gb) |
| *Solanum lycopersicum* | 298,306 | 1,518 | 3,399,630 | 2X50: 196.9M (19.5 Gb)  1X50: 110.2M (5.4 Gb) |
| *Vitis vinifera* | 446,668 | 978 | NA | 2X51: 147.1M (15 Gb)  2X100: 354.8M (71 Gb) |

**Supplementary Table 3. RNA-seq tissues types and download sources.**

| Species | RNA-seq Tissue | Source of RNA-seq Data Collection |
| --- | --- | --- |
| *Amborella trichopoda* | Apical meristem, flower, flower buds, fruit, leaves, roots, shoot, and whole plant normalized | Data from oneKP project (<http://www.onekp.com>): Sample code "URDJ".  AAGP website (<http://ancangio.uga.edu/illumina-data>): AmTr_ap_mer, AmTr_fem_bud, AmborellaWPN-1, and AmborellaWPN-2.  Unpublished data from *Amborella* Genome Project (<http://www.amborella.org/>) and Claude dePamphilis (cwd3@psu.edu) |
| *Arabidopsis thaliana* | Floral bud, flower, root, seed, and siliques. | NCBI SRA Accession: SRR314813, SRR314814, SRR314815, SRR360147, SRR360152, SRR360153, SRR360154, SRR360205, SRR391051, SRR391052, SRR505743, SRR505744, SRR505745, SRR505746 |
| *Glycine max* | Floral buds, flower, leaves, nodules, pod, root, root hairs, SAM, seed, and stem. | NCBI SRA Accession: SRR203366, SRR203367  Additional RNA-seq data sets were provided from Gary Stacey (University of Missouri, USA) and Jeremy Schmutz (DOE Joint Genome Institute, USA). |
| *Medicago truncatula* | Multiple tissues pooled, root, and seedling. | NCBI SRA Accession (Illumina): SRR670348, SRR670349, SRR670350, SRR670345, SRR670346, SRR670347, SRR670351, SRR670352, SRR670353, SRR670354, SRR670355, SRR670356, SRR670357, SRR670358, SRR670383, SRR670400, SRR670403, SRR670404.  Multiple tissues pooled RNA-seq data is from (Tang et al., 2014). |

Supplemental Table 3. Continued

| Species | RNA-seq Tissue | Source of RNA-seq Data Collection |
| --- | --- | --- |
| *Oryza sativa* | Leaf, panicle, root, and young ear | NCBI SRA Accession (Illumina): DRR013722, DRR013723, SRR606414, SRR606408, SRR037739, SRR037738, SRR072076, and SRR072077 |
| *Phaseolus vulgaris* | Flower buds, flowers, leaves, nodules, pods, roots, stem, and trifoliates. | Collaborator Scott Jackson provided RNA-seq data, and can be accessed via following url.  <ftp://ftp.jgi-psf.org/pub/compgen/phytozome/v9.0/Pvulgaris/related_files/expression/bam/> |
| *Populus trichocarpa* | Buds, cambium/phloem, flowers, leaves, petiole, roots, seeds, suckers, and twigs. | The *P. tremula* RNA-Seq expression atlas dataset was provided by the Umeå Plant Science Centre (personal communication with Nathaniel Street, Umeå University, Sweden) and is available from the ENA repository under accession ID ERP004398. The data is also made available for visualisation of expression within the various samples at the PopGenIE resource (Sjödin et al., 2009). |

Supplemental Table 3. Continued

| Species | RNA-seq Tissue | Source of RNA-seq Data Collection |
| --- | --- | --- |
| *Solanum lycopersicum* | Flower, flower bud, fruit developmental stages, leaf, meristem, pericarp of fruit, pollen, pollinated style, root, stem, and unpollinated style. | NCBI SRA Accession (454 GS FLX Titanium): SRR363116, SRR363117, SRR363118, SRR363119, SRR363120, SRR363121, SRR363122, SRR088753, SRR088751, SRR088749, SRR088748, SRR088747, SRR088745, SRR088744, SRR088743, SRR088742, SRR088741, SRR088740, SRR088739, SRR088738, SRR088737, SRR088736, SRR088735, SRR088734, SRR088733, SRR088732  NCBI SRA Accession (Illumina): SRR404309, SRR404310, SRR404311, SRR404312, SRR404313, SRR404314, SRR404315, SRR404316, SRR404317, SRR404318, SRR404319, SRR404320, SRR404321, SRR404322, SRR404324, SRR404325, SRR404326, SRR404327, SRR404328, SRR404329, SRR404331, SRR404333, SRR404334, SRR404336, SRR404338, SRR404339, SRR412747, SRR412748, SRR567999, SRR568000 |
| *Vitis vinifera* | Fruit, leaves, and multiple tissues pooled. | NCBI SRA Accession (Illumina): SRR519449, SRR519450, SRR519451, SRR519452, SRR519453, SRR519454, SRR519455, SRR519456, SRR520374, SRR520376, SRR520378, SRR520379, SRR520380, SRR520381, SRR520382, SRR520384, SRR520385, SRR520386, SRR520387, SRR520388, SRR522298, SRR522471, SRR522472, SRR522473, SRR522474, SRR522475, SRR522477, SRR522478, SRR522479, SRR522484 |

**Supplementary Table 4. Intron sizes used while performing transcriptome alignments and assemblies.**

| Species | Mean exon size | 99^th^ Percentile of maximum intron size per gene | Intron sizes used in transcriptome alignments and assemblies  (Mean exon sizes + 99^th^ percentile) |
| --- | --- | --- | --- |
| *Amborella trichopoda* | 517 | 26,957 | 27,474 |
| *Arabidopsis thaliana* | 753 | 1,586 | 2,339 |
| *Glycine max* | 868 | 8,099 | 8,967 |
| *Medicago truncatula* | 673 | 4,625 | 5,556 |
| *Oryza sativa* | 344 | 5,625 | 6,623 |
| *Phaseolus vulgaris* | 815 | 6,081 | 6,896 |
| *Populus trichocarpa* | 826 | 4,590 | 5,416 |
| *Solanum lycopersicum* | 627 | 6,731 | 7,358 |
| *Vitis vinifera* | 636 | 20,134 | 20,770 |

**Supplementary Table 5. Conserved AS events retention and loss categories among WGD orthologs between common bean (CB) and soybean (SB) and their conservation with at least one other angiosperms.**

|  | 1:2 | | 1:1 | | 0:2 | | 1:0 | | 0:1 | |
| --- | --- | --- | --- | --- | --- | --- | --- | --- | --- | --- |
|  | CB | SB | CB | SB | CB | SB | CB | SB | CB | SB |
| AS Events | 1907 | 3914 | 2726 | 2758 | NA | 5708 | 8497 | NA | NA | 21816 |
| Conserved with one other species apart from CB and SB | 1252  (65.7%) | 2451  (62.6%) | 1445  (53%) | 1374  (49.8%) | NA | 2460  (43.1%) | 2574  (30.3%) | NA | NA | 5378  (24.7%) |

**Supplementary Table 6. GO Enrichment Analysis (Fisher's Exact Test) with BLAST2GO of genes having conserved AS events conserved across at least six angiosperms.** In Category column, “C” is Cellular Component, “F” is Molecular Function, and “P” is Biological Process.

| GO-ID | Term | Category | P-Value | Over/Under |
| --- | --- | --- | --- | --- |
| GO:0003824 | catalytic activity | F | 1.42E-47 | over |
| GO:0016301 | kinase activity | F | 1.19E-35 | over |
| GO:0016772 | transferase activity, transferring phosphorus-containing groups | F | 4.29E-32 | over |
| GO:0043167 | ion binding | F | 8.64E-28 | over |
| GO:0071944 | cell periphery | C | 1.66E-26 | over |
| GO:0016740 | transferase activity | F | 2.14E-25 | over |
| GO:0005886 | plasma membrane | C | 1.42E-24 | over |
| GO:0016020 | Membrane | C | 1.49E-24 | over |
| GO:0044238 | primary metabolic process | P | 3.74E-24 | over |
| GO:0071704 | organic substance metabolic process | P | 3.77E-24 | over |
| GO:0005488 | Binding | F | 1.47E-20 | over |
| GO:0036211 | protein modification process | P | 8.18E-19 | over |
| GO:0006464 | cellular protein modification process | P | 8.18E-19 | over |
| GO:0009987 | cellular process | P | 1.05E-18 | over |
| GO:0043412 | macromolecule modification | P | 1.18E-18 | over |
| GO:0044699 | single-organism process | P | 2.10E-18 | over |
| GO:0022857 | transmembrane transporter activity | F | 1.13E-17 | over |
| GO:0055085 | transmembrane transport | P | 3.34E-16 | over |
| GO:0008152 | metabolic process | P | 4.04E-16 | over |
| GO:0050789 | regulation of biological process | P | 1.03E-15 | Over |
| GO:0044763 | single-organism cellular process | P | 1.34E-15 | over |
| GO:0005215 | transporter activity | F | 1.38E-15 | over |
| GO:0007165 | signal transduction | P | 1.54E-15 | over |
| GO:0051716 | cellular response to stimulus | P | 1.54E-15 | over |
| GO:0050794 | regulation of cellular process | P | 1.56E-15 | Over |
| GO:0023052 | Signaling | P | 9.42E-15 | over |
| GO:0007154 | cell communication | P | 9.42E-15 | over |
| GO:0044700 | single organism signaling | P | 9.42E-15 | over |
| GO:0016787 | hydrolase activity | F | 1.18E-13 | over |
| GO:0016798 | hydrolase activity, acting on glycosyl bonds | F | 2.56E-13 | over |
| GO:0065007 | biological regulation | P | 2.80E-13 | over |

Supplementary Table 6. Continued

| GO-ID | Term | Category | P-Value | Over/Under |
| --- | --- | --- | --- | --- |
| GO:0044267 | cellular protein metabolic process | P | 1.32E-12 | over |
| GO:0019538 | protein metabolic process | P | 1.61E-12 | over |
| GO:0004871 | signal transducer activity | F | 2.51E-12 | over |
| GO:0060089 | molecular tranphotosducer activity | F | 2.51E-12 | over |
| GO:0044765 | single-organism transport | P | 5.30E-12 | over |
| GO:0050896 | response to stimulus | P | 1.13E-11 | over |
| GO:0043170 | macromolecule metabolic process | P | 3.19E-11 | over |
| GO:0044260 | cellular macromolecule metabolic process | P | 4.11E-11 | over |
| GO:0005975 | carbohydrate metabolic process | P | 8.85E-11 | Over |
| GO:0051234 | establishment of localization | P | 2.83E-10 | over |
| GO:0006810 | Transport | P | 2.83E-10 | over |
| GO:0051179 | Localization | P | 2.85E-10 | over |
| GO:0008219 | cell death | P | 8.49E-09 | over |
| GO:0016265 | Death | P | 8.49E-09 | over |
| GO:0006397 | mRNA processing | P | 3.70E-08 | over |
| GO:0016071 | mRNA metabolic process | P | 3.70E-08 | over |
| GO:0006396 | RNA processing | P | 4.07E-08 | over |
| GO:0044237 | cellular metabolic process | P | 6.55E-08 | over |
| GO:0002376 | immune system process | P | 3.17E-07 | over |
| GO:0016070 | RNA metabolic process | P | 9.77E-07 | over |
| GO:0044710 | single-organism metabolic process | P | 2.21E-06 | over |
| GO:0061024 | membrane organization | P | 4.16E-06 | over |
| GO:0006950 | response to stress | P | 2.34E-05 | over |
| GO:0042578 | phosphoric ester hydrolase activity | F | 2.40E-05 | over |
| GO:0016791 | phosphatase activity | F | 2.40E-05 | over |
| GO:0016757 | transferase activity, transferring glycosyl groups | F | 7.38E-05 | over |
| GO:0016491 | oxidoreductase activity | F | 7.53E-05 | over |
| GO:0021700 | developmental maturation | P | 8.66E-05 | over |
| GO:0016788 | hydrolase activity, acting on ester bonds | F | 1.66E-04 | over |
| GO:0003723 | RNA binding | F | 6.57E-04 | over |
| GO:0007568 | Aging | P | 6.66E-04 | over |
| GO:0044281 | small molecule metabolic process | P | 1.98E-03 | over |
| GO:0019439 | aromatic compound catabolic process | P | 2.55E-03 | over |

Supplementary Table 6. Continued

| GO-ID | Term | Category | P-Value | Over/Under |
| --- | --- | --- | --- | --- |
| GO:1901361 | organic cyclic compound catabolic process | P | 2.55E-03 | over |
| GO:0044270 | cellular nitrogen compound catabolic process | P | 2.55E-03 | over |
| GO:0044248 | cellular catabolic process | P | 2.55E-03 | over |
| GO:0034655 | nucleobase-containing compound catabolic process | P | 2.55E-03 | over |
| GO:0046700 | heterocycle catabolic process | P | 2.55E-03 | over |
| GO:0003729 | mRNA binding | F | 2.56E-03 | over |
| GO:0044822 | poly(A) RNA binding | F | 2.56E-03 | over |
| GO:1901575 | organic substance catabolic process | P | 2.58E-03 | over |
| GO:0019748 | secondary metabolic process | P | 3.70E-03 | over |

**Supplementary Table 7. GO Enrichment Analysis (Fisher's Exact Test) with BLAST2GO of genes having AS events conserved in 1:2 categories of soybean.** In Category column, “C” is Cellular Component, “F” is Molecular Function, and “P” is Biological Process.

| GO-ID | Term | Category | P-Value | Over/Under |
| --- | --- | --- | --- | --- |
| GO:0003676 | nucleic acid binding | F | 1.30E-08 | over |
| GO:0005634 | Nucleus | C | 3.04E-05 | over |
| GO:0004518 | nuclease activity | F | 2.03E-04 | over |
| GO:0016788 | hydrolase activity, acting on ester bonds | F | 2.03E-04 | over |
| GO:0003723 | RNA binding | F | 2.11E-04 | over |
| GO:0097159 | organic cyclic compound binding | F | 1.31E-03 | over |
| GO:1901363 | heterocyclic compound binding | F | 1.31E-03 | over |
| GO:0009991 | response to extracellular stimulus | P | 2.25E-03 | over |
| GO:0043231 | intracellular membrane-bounded organelle | C | 3.64E-03 | over |
| GO:0043227 | membrane-bounded organelle | C | 3.64E-03 | over |

**Supplementary Table 8. *Vitis vinifera* cv. Corvina samples pooled for RNA-seq run (23 Gb; 114.7 M; 2X100) by** (Venturini et al., 2013)**.**

| Sample / organ | Developmental stages collected | Total Samples |
| --- | --- | --- |
| Bud | first season latent bud, winter/dormant bud, bud scales opening, wooly-cotton bud (green showing), bud after bud burst (shoot with one leaf visible) | 5 |
| Inflorescence | young inflorescence, well developed inflorescence (single flower separated), flowering begins (30% caps off) | 3 |
| Tendril | young tendril, mature tendril | 2 |
| Leaf | young leaf (5 leaves separated), mature leaf, senescencing leaf (beginning of leaf fall) | 3 |
| Berry (whole) | fruit set | 1 |
| Berry Skin | post fruit set, véraison, pre-ripening, ripening, post harvest withering process I, II, and III | 7 |
| Berry Flesh | post fruit set, véraison, pre-ripening, ripening, post harvest withering process I, II, and III | 7 |
| Seed | fruit set, post fruit set, véraison, full mature seed (pool from pre-ripening and ripening) | 4 |
| Rachis | fruit set, post fruit set, véraison, pre-ripening, and ripening | 5 |
| Stem | green stem (from the cane), woody stem (complete cane maturation) | 2 |
| Root (“in vitro”) | Pool | 1 |
| Seedling | pool (from 3 different stages of seedling) | 1 |
| Anther | pool (from 2 different stages of flower development) | 1 |
| Pollen | Pool | 1 |
| Carpel | pool (from 2 different stages of flower development) | 1 |
| Petal | pool (from 2 different stages of flower development) | 1 |
| Total |  | 45 |

**Supplementary Table 9. AS events of MADS box gene family type I.**

| AS Type | | Amborella | Arabidopsis |
| --- | --- | --- | --- |
| AltA | Events (%) | 1 (20.0%) | 2 (33.3%) |
|  | Genes (%) | 1 (25%) | 2 (15.4%) |
| AltD | Events (%) | 0 (0%) | 1 (16.7%) |
|  | Genes (%) | 0 (0%) | 1 (7.7%) |
| ExonS | Events (%) | 2 (40.0%) | 0 (0%) |
|  | Genes (%) | 2 (50%) | 0 (0%) |
| IntronR | Events (%) | 2 (40.0%) | 3 (50.0%) |
|  | Genes (%) | 1 (25%) | 2 (15.4%) |
| Total | Events | 5 | 6 |
|  | Genes with AS (%) | 4 (100%) | 4 (30.8%) |
|  | Genes | 12 | 57 |
|  | Expressed Multi-exonic Genes | 4 | 13 |

Note: Genes (%) above is calculated based on total expressed multi-exonic genes.

**Supplementary Table 10. AS events of MADS box gene family type II.**

| AS Type | | Amborella | Arabidopsis | Rice | Poplar |
| --- | --- | --- | --- | --- | --- |
| AltA | Events (%) | 46 (15.3%) | 23 (20.2%) | 17 (23.9%) | 15 (25.0%) |
|  | Genes (%) | 20 (100%) | 15 (33.3%) | 11 (30.6%) | 10 (25.6%) |
| AltD | Events (%) | 29 (9.7%) | 15 (13.2%) | 10 (14.1%) | 8 (13.3%) |
|  | Genes (%) | 13 (65%) | 11 (24.4%) | 9 (25%) | 6 (15.4%) |
| ExonS | Events (%) | 27 (9.0%) | 7 (6.1%) | 7 (9.9%) | 6 (10.0%) |
|  | Genes (%) | 11 (55%) | 5 (11.1%) | 4 (11.1%) | 4 (10.3%) |
| IntronR | Events (%) | 198 (66.0%) | 69 (60.5%) | 37 (52.1%) | 31 (51.7%) |
|  | Genes (%) | 8 (40%) | 26 (57.8%) | 15 (41.7%) | 16 (41%) |
| Total | Events | 300 | 114 | 71 | 60 |
|  | Genes with AS (%) | 20 (100%) | 32 (71.1%) | 24 (66.7%) | 22 (56.4%) |
|  | Genes | 23 | 45 | 37 | 44 |
|  | Expressed Multi-exonic Genes | 20 | 45 | 36 | 39 |

Note: Genes (%) above is calculated based on total expressed multi-exonic genes.

**Supplementary Table 11. AS events of SR Protein gene family.**

| AS Type | | Arabidopsis | Soybean | Rice | Poplar |
| --- | --- | --- | --- | --- | --- |
| AltA | Events (%) | 17 (21.8%) | 31 (22.5%) | 26 (22.0%) | 24 (23.5%) |
|  | Genes (%) | 11 (61.1%) | 17 (73.9%) | 20 (90.9%) | 13 (68.4%) |
| AltD | Events (%) | 11 (14.1%) | 25 (18.1%) | 12 (10.2%) | 8 (7.8%) |
|  | Genes (%) | 10 (55.6%) | 16 (69.6%) | 9 (40.9%) | 7 (36.8%) |
| ExonS | Events (%) | 10 (12.8%) | 20 (14.5%) | 18 (15.3%) | 13 (12.7%) |
|  | Genes (%) | 6 (33.3%) | 13 (56.5%) | 13 (61.9%) | 9 (47.4%) |
| IntronR | Events (%) | 40 (51.3%) | 62 (44.9%) | 62 (52.5%) | 57 (55.9%) |
|  | Genes (%) | 15 (83.3%) | 18 (78.3%) | 19 (86.4%) | 16 (84.2%) |
| Total | Events | 78 | 138 | 118 | 102 |
|  | Genes with AS (%) | 16 (88.9%) | 22 (95.7%) | 21 (95.5%) | 17 (89.5%) |
|  | Genes | 19 | 26 | 24 | 20 |
|  | Expressed Multi-exonic Genes | 18 | 23 | 22 | 19 |

Note: Genes (%) above is calculated based on total expressed multi-exonic genes.

## Supplementary Figures


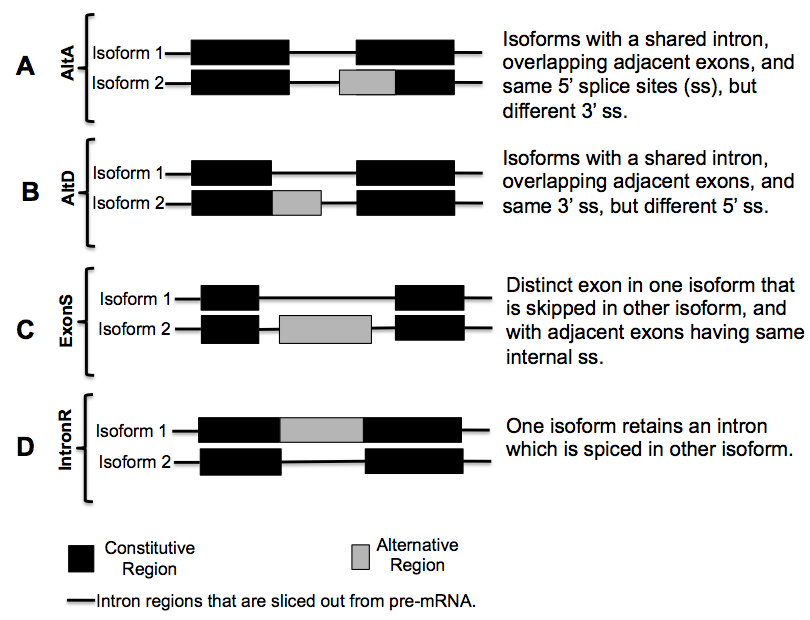


**Supplementary Figure 1. Types of AS events.** (A) Alternative acceptor or Alternative 3’ splice site (ss). (B) Alternative donor or Alternative 5’ ss. (C) Exon skip. (D) Intron retention.


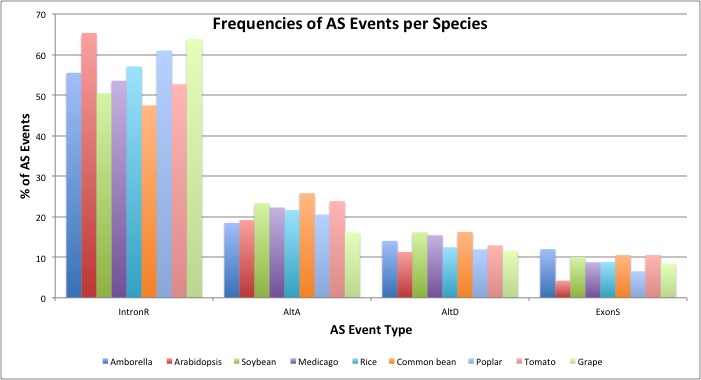


**Supplementary Figure 2****. Frequencies of AS events.**


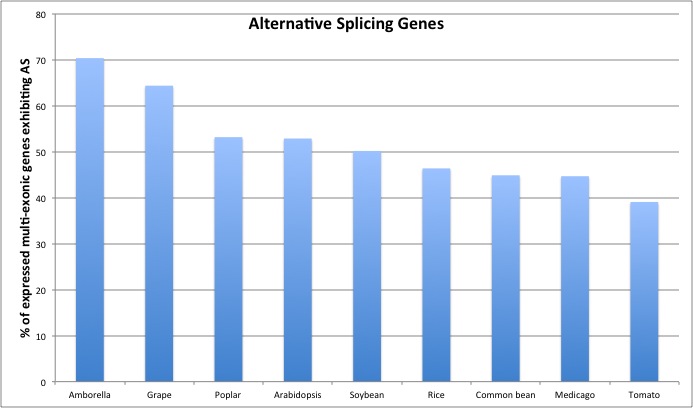


**Supplementary Figure 3.**  **Frequencies of AS in expressed multi-exonic genes.**


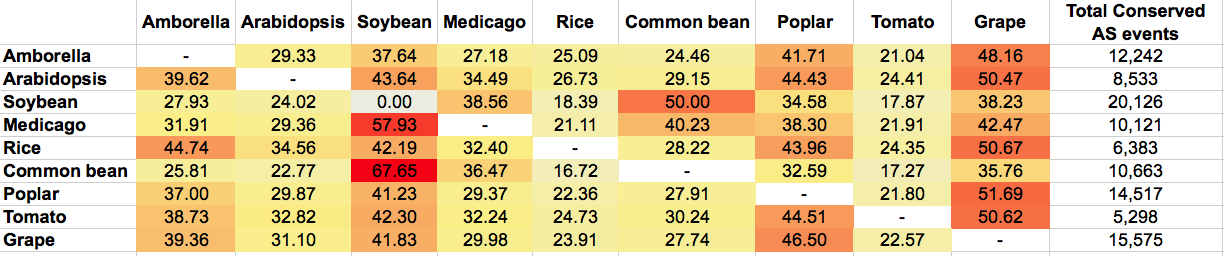


Supplementary Figure 4. Percentages of conserved AS events shared between species. The way to read this table is for example, 28.33% of 12,242 conserved AS events of *Amborella* are conserved with *Arabidopsis.* Percentage of events are with species in the rows.


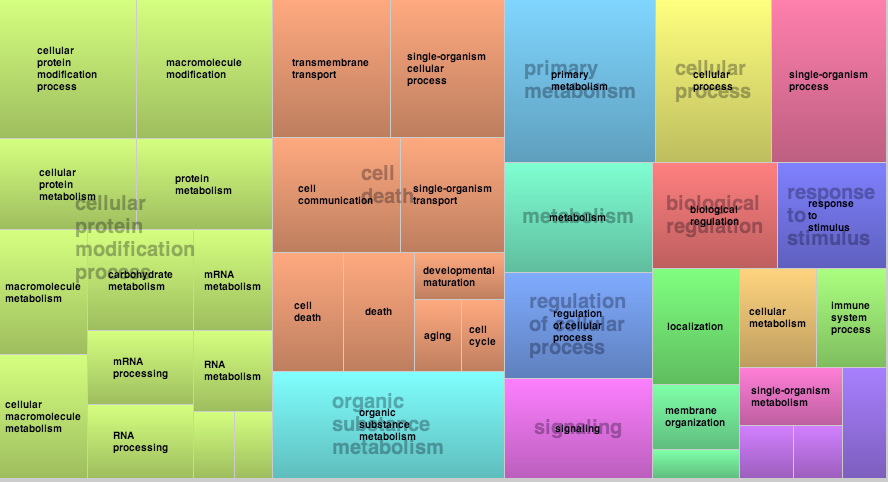


**Supplementary Figure 5. Visualization of enriched GO terms associated with “Biological Process” category of genes having conserved AS events conserved across at least six angiosperms** (Supek et al., 2011) **.**


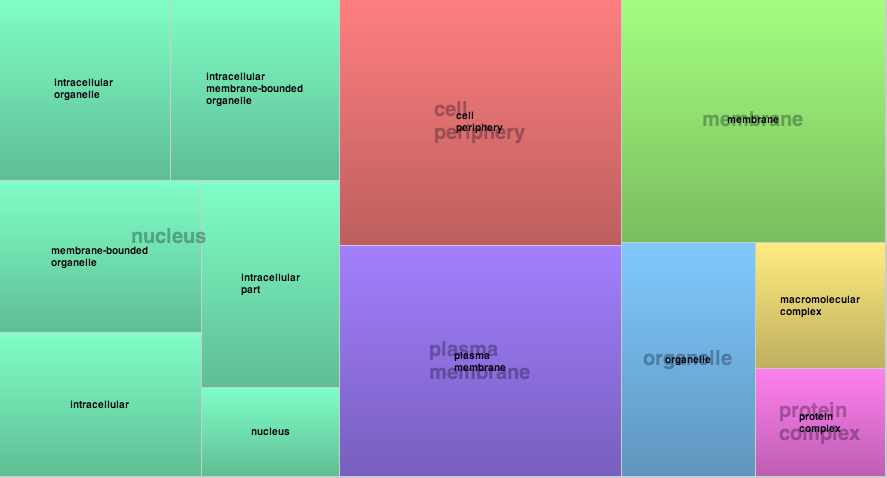


**Supplementary Figure 6. Visualization of enriched GO terms associated with “Cellular Component” category of genes having conserved AS events conserved across at least six angiosperms** (Supek et al., 2011)**.**


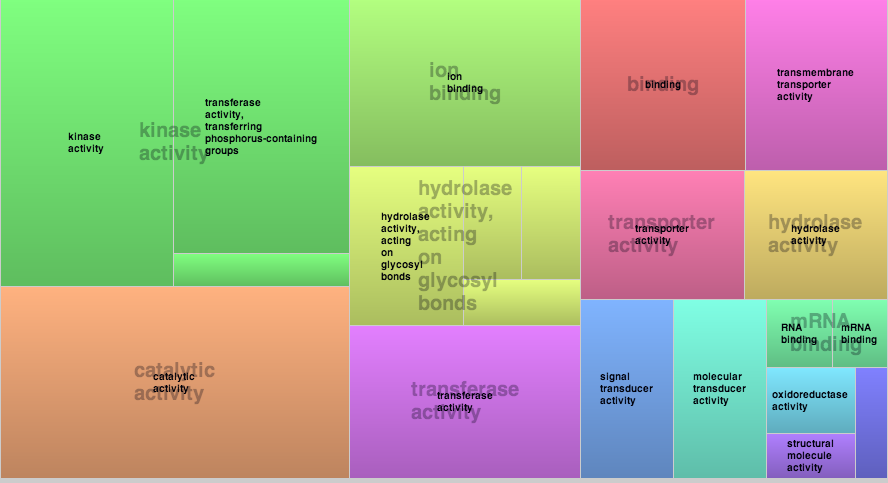


**Supplementary Figure 7. Visualization of enriched GO terms associated with “Molecular Function” category of genes having conserved AS events conserved across at least six angiosperms** (Supek et al., 2011)**.**


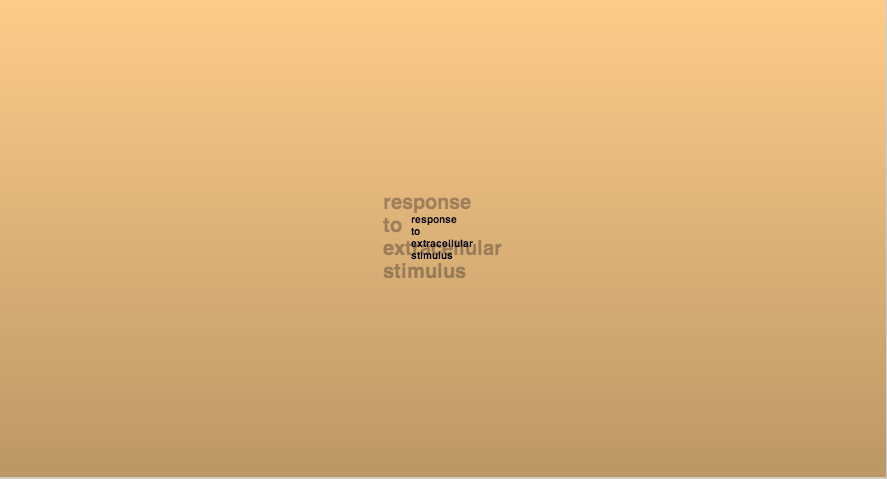


**Supplementary Figure 8. Visualization of enriched GO terms associated with “Biological Process” category of genes having conserved AS events in two WGD paralogs of soybean and their corresponding ortholog in common bean** (Supek et al., 2011)**.**


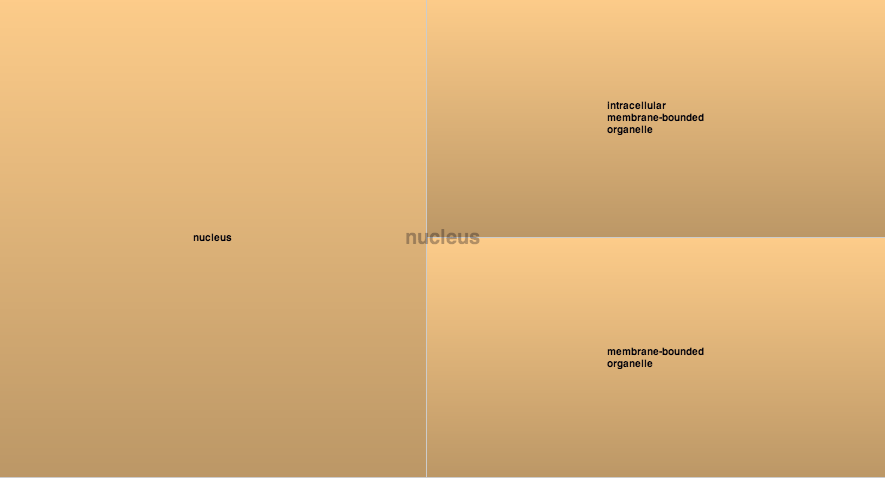


**Supplementary Figure 9. Visualization of enriched GO terms associated with “Cellular Component” category of genes having conserved AS events in two WGD paralogs of soybean and their corresponding ortholog in common bean** (Supek et al., 2011)**.**


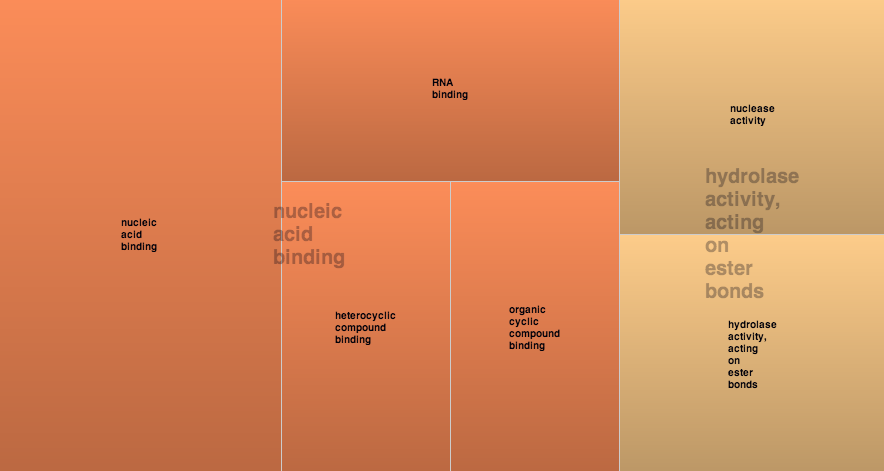


**Supplementary Figure 10. Visualization of enriched GO terms associated with “Molecular Function” category of genes having conserved AS events in two WGD paralogs of soybean and their corresponding ortholog in common bean** (Supek et al., 2011)**.**


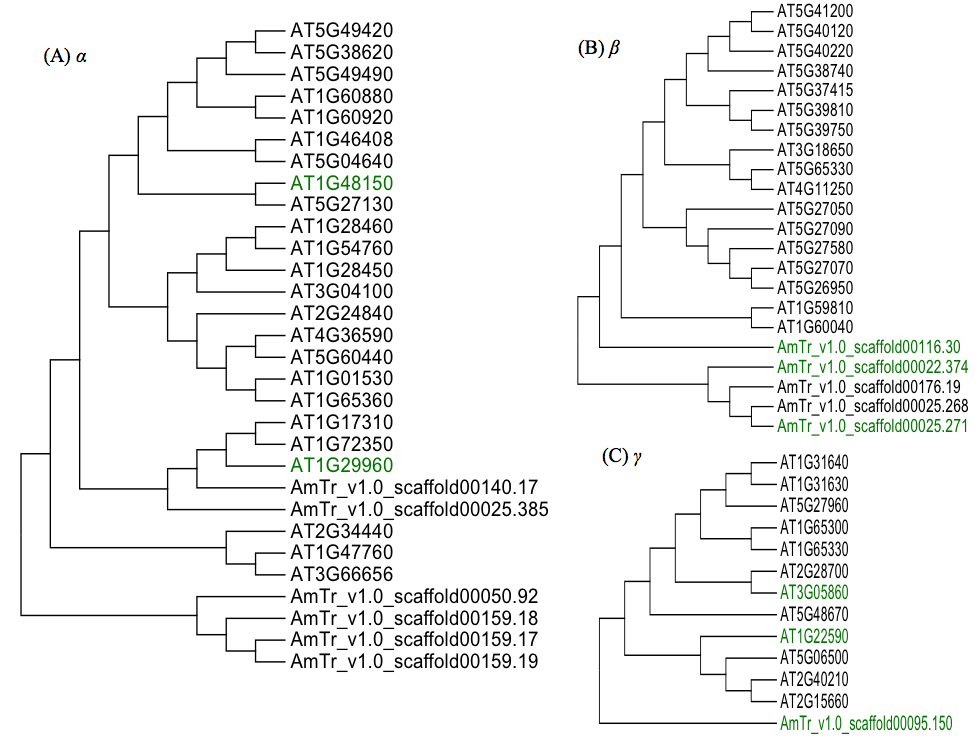


**Supplementary Figure 11. Gene trees for subfamilies of type I MADS-box in *Amborella trichopoda* and *Arabidopsis thaliana*.** The trees were constructed with maximum likelihood method. Gene name in green indicate evidence for alternative splicing event(s).


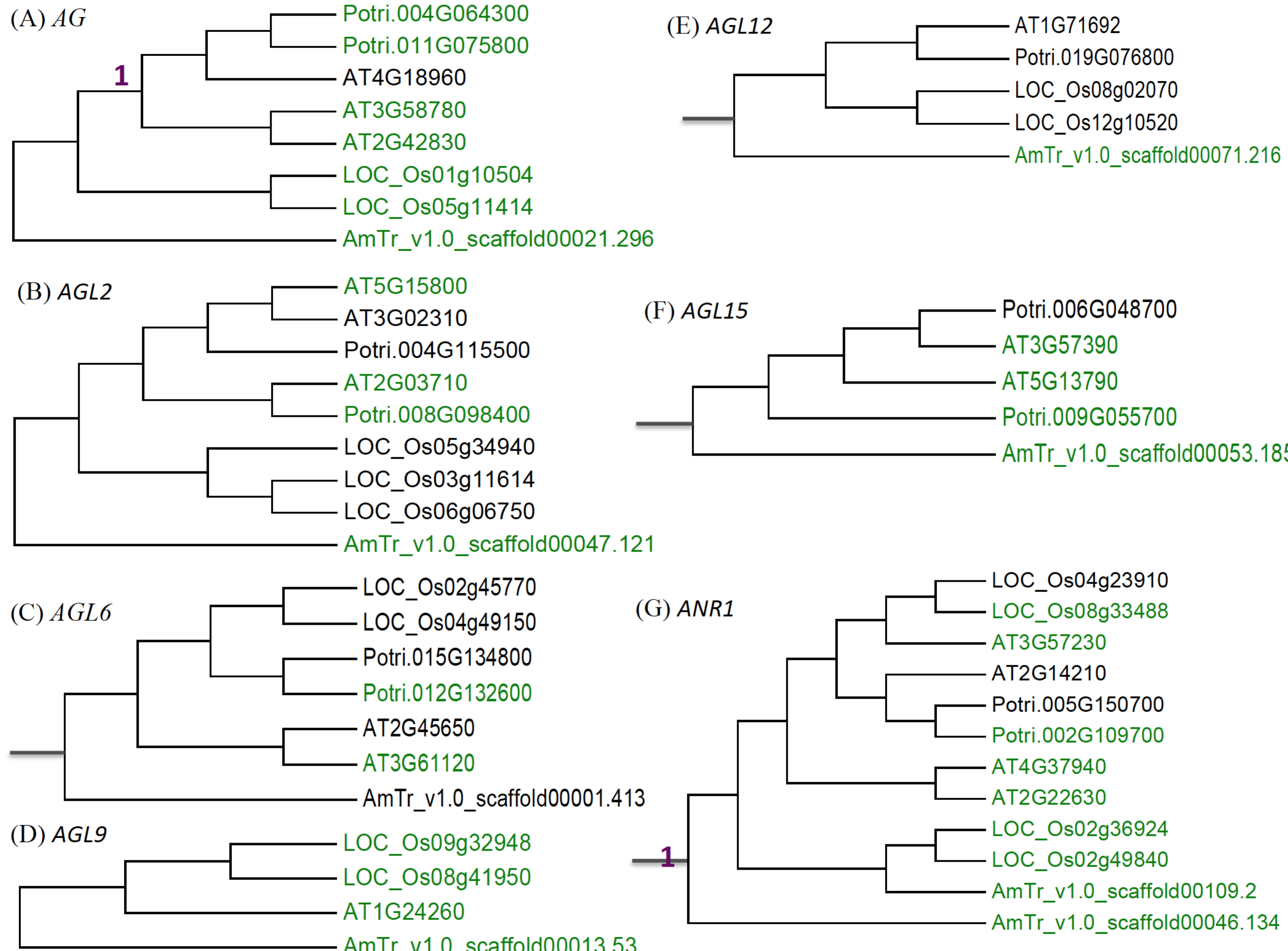


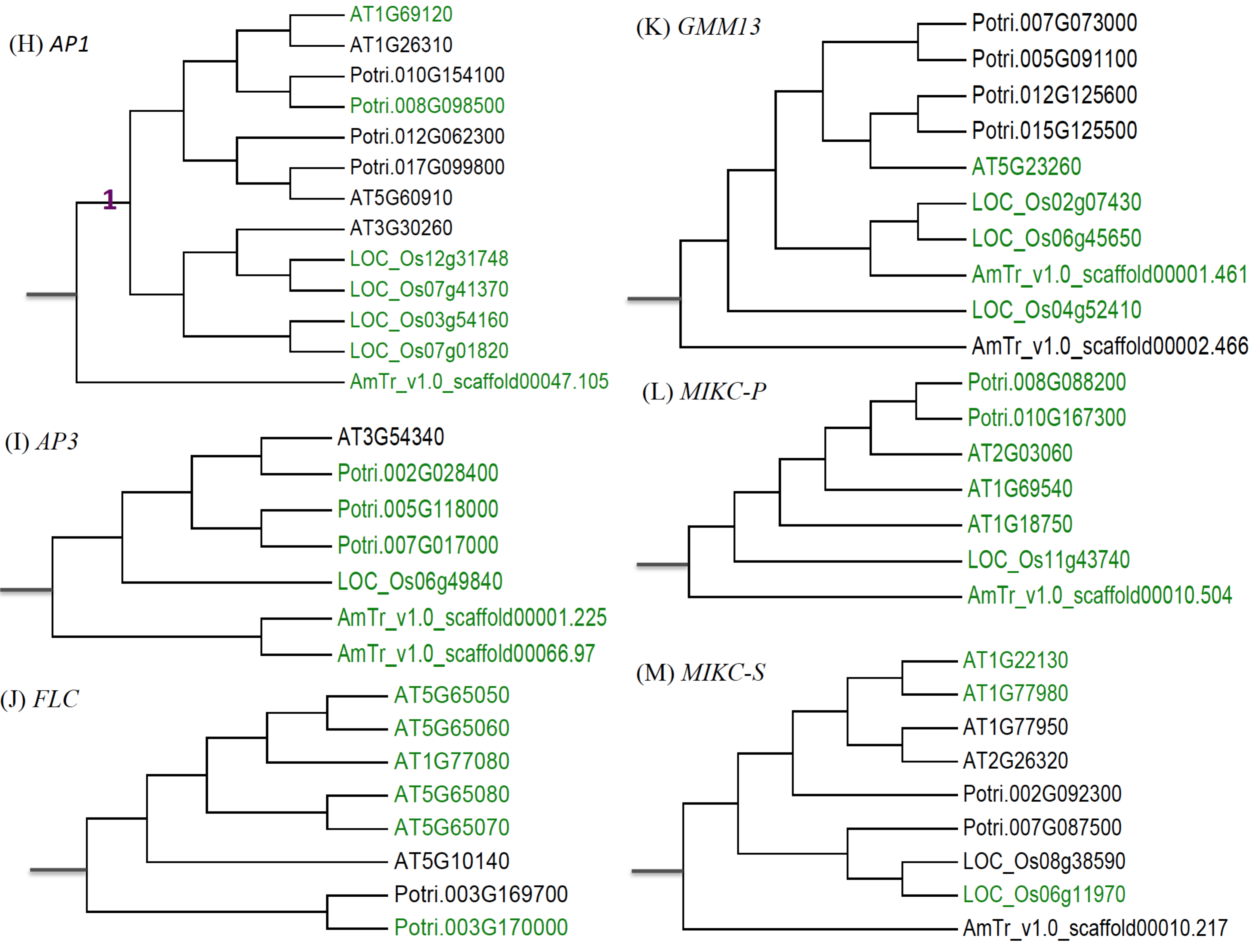


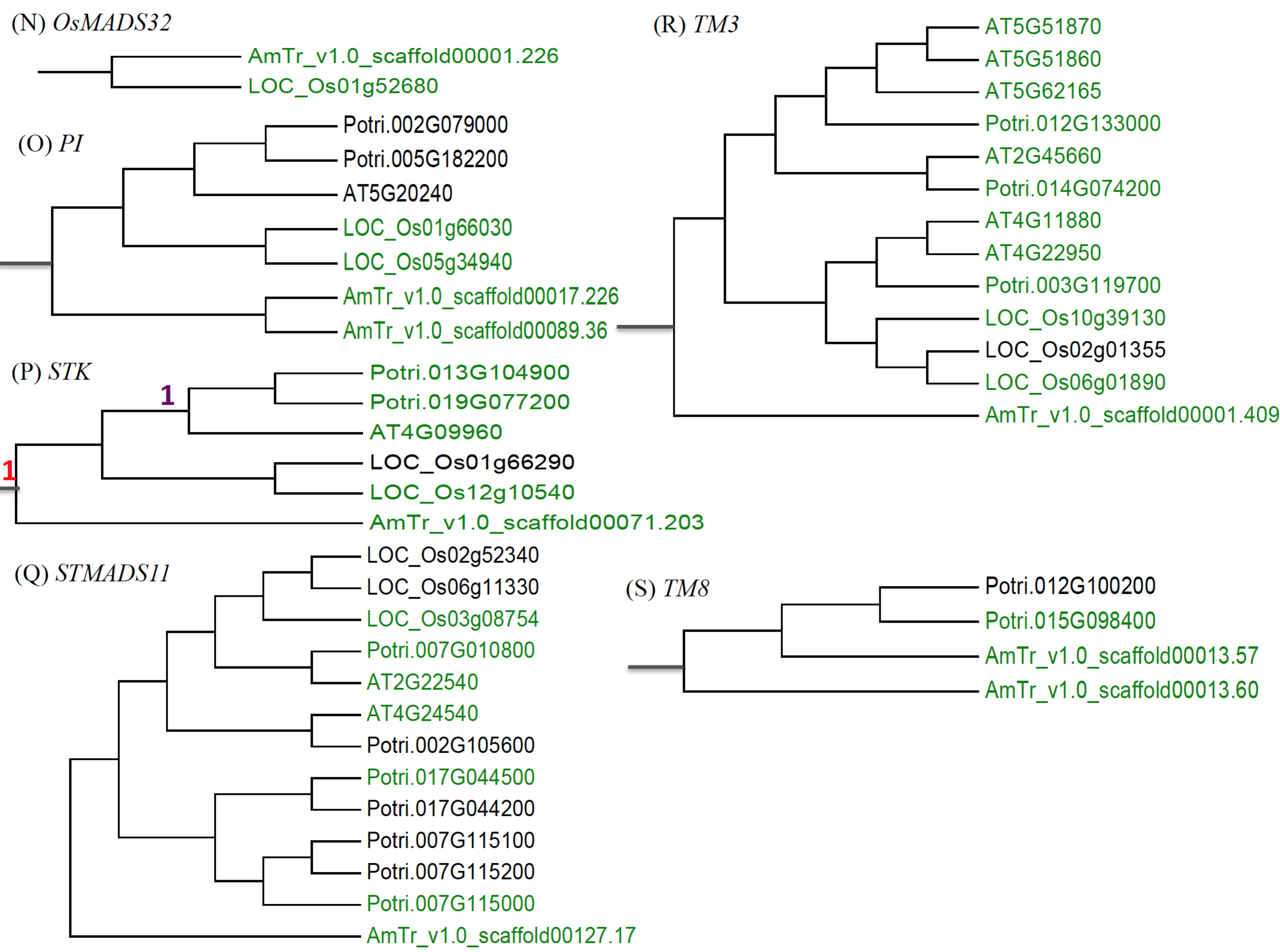


**Supplementary Figure 12. Gene trees for subfamilies of type II MADS-box in *Amborella trichopoda*, *Arabidopsis thaliana*, *Populus trichocarpa* and *Oryza sativa*.**The trees were constructed with maximum likelihood method. Gene name in green indicate evidence for alternative splicing event(s). Number above branch indicates total alternative splicing events that are conserved between members of corresponding clade. Numbers are depicted in red and purple representing alternative acceptor and intron retention events respectively.

1. **References**

Altschul, S. F., Gish, W., Miller, W., Myers, E. W., and Lipman, D. J. (1990). Basic local alignment search tool. *J. Mol. Biol.* 215, 403–410.

Altschul, S. F., Madden, T. L., Schäffer, A. A., Zhang, J., Zhang, Z., Miller, W., and Lipman, D. J. (1997). Gapped BLAST and PSI-BLAST: a new generation of protein database search programs. *Nucleic Acids Res.* 25, 3389–3402. doi:10.1093/nar/25.17.3389.

Amborella Genome Project (2013). The Amborella Genome and the Evolution of Flowering Plants. *Science.* 342. doi:10.1126/science.1241089.

Chamala, S., Chanderbali, A. S., Der, J. P., Lan, T., Walts, B., Albert, V. A., Leebens-Mack, J., Rounsley, S., Schuster, S. C., and Wing, R. A. (2013). Assembly and Validation of the Genome of the Nonmodel Basal Angiosperm Amborella. *Science.* 342, 1516–1517. doi:10.1126/science.1241130.

Conesa, A., Götz, S., García-Gómez, J. M., Terol, J., Talón, M., and Robles, M. (2005). Blast2GO: a universal tool for annotation, visualization and analysis in functional genomics research. *Bioinformatics* 21, 3674–3676. doi:10.1093/bioinformatics/bti610.

Grabherr, M. G., Haas, B. J., Yassour, M., Levin, J. Z., Thompson, D. A., Amit, I., Adiconis, X., Fan, L., Raychowdhury, R., and Zeng, Q. (2011). Full-length transcriptome assembly from RNA-Seq data without a reference genome. *Nat. Biotechnol.* 29, 644–652. doi:10.1038/nbt.1883.

Haas, B. J., Delcher, A. L., Mount, S. M., Wortman, J. R., Smith Jr, R. K., Hannick, L. I., Maiti, R., Ronning, C. M., Rusch, D. B., and Town, C. D. (2003). Improving the Arabidopsis genome annotation using maximal transcript alignment assemblies. *Nucleic Acids Res.* 31, 5654–5666. doi:10.1093/nar/gkg770.

Haas, B. J., Papanicolaou, A., Yassour, M., Grabherr, M., Blood, P. D., Bowden, J., Couger, M. B., Eccles, D., Li, B., and Lieber, M. (2013). De novo transcript sequence reconstruction from RNA-seq using the Trinity platform for reference generation and analysis. *Nat. Protoc.* 8, 1494–1512. doi:10.1038/nprot.2013.084.

Haas, B. J., Zeng, Q., Pearson, M. D., Cuomo, C. A., and Wortman, J. R. (2011). Approaches to fungal genome annotation. *Mycology* 2, 118–141.

Jaillon, O., Aury, J. M., Noel, B., Policriti, A., Clepet, C., Casagrande, A., Choisne, N., Aubourg, S., Vitulo, N., Jubin, C., et al. (2007). The grapevine genome sequence suggests ancestral hexaploidization in major angiosperm phyla. *Nature* 449, 463–467. doi:10.1038/Nature06148.

Kent, W. J. (2002). BLAT—the BLAST-like alignment tool. *Genome Res.* 12, 656–664. doi:10.1101/gr.229202.

Kim, D., Pertea, G., Trapnell, C., Pimentel, H., Kelley, R., and Salzberg, S. L. (2013). TopHat2: accurate alignment of transcriptomes in the presence of insertions, deletions and gene fusions. *Genome Biol.* 14, R36. doi:10.1186/gb-2013-14-4-r36.

Lohse, M., Bolger, A. M., Nagel, A., Fernie, A. R., Lunn, J. E., Stitt, M., and Usadel, B. (2012). RobiNA: a user-friendly, integrated software solution for RNA-Seq-based transcriptomics. *Nucleic Acids Res.* 40, W622–W627. doi:Doi 10.1093/Nar/Gks540.

Martin, M. (2011). Cutadapt removes adapter sequences from high-throughput sequencing reads. *EMBnet. J.* 17, pp. 10–12. doi:10.14806/ej.17.1.200.

Ouyang, S., Zhu, W., Hamilton, J., Lin, H., Campbell, M., Childs, K., Thibaud-Nissen, F., Malek, R. L., Lee, Y., and Zheng, L. (2007). The TIGR rice genome annotation resource: improvements and new features. *Nucleic Acids Res.* 35, D883–D887. doi:10.1093/nar/gkl976.

Rhind, N., Chen, Z., Yassour, M., Thompson, D. A., Haas, B. J., Habib, N., Wapinski, I., Roy, S., Lin, M. F., and Heiman, D. I. (2011). Comparative functional genomics of the fission yeasts. *Science.* 332, 930–936. doi:10.1126/science.1203357.

Sato, S., Tabata, S., Hirakawa, H., Asamizu, E., Shirasawa, K., Isobe, S., Kaneko, T., Nakamura, Y., Shibata, D., Aoki, K., et al. (2012). The tomato genome sequence provides insights into fleshy fruit evolution. *Nature* 485, 635–641. doi:Doi 10.1038/Nature11119.

Schmutz, J., Cannon, S. B., Schlueter, J., Ma, J., Mitros, T., Nelson, W., Hyten, D. L., Song, Q., Thelen, J. J., and Cheng, J. (2010). Genome sequence of the palaeopolyploid soybean. *Nature* 463, 178–183. doi:10.1038/nature08670.

Sjödin, A., Street, N. R., Sandberg, G., Gustafsson, P., and Jansson, S. (2009). The Populus Genome Integrative Explorer (PopGenIE): a new resource for exploring the Populus genome. *New Phytol.* 182, 1013–1025. doi:10.1111/j.1469-8137.2009.02807.x.

Supek, F., Bošnjak, M., Škunca, N., and Šmuc, T. (2011). REVIGO summarizes and visualizes long lists of gene ontology terms. *PLoS One* 6, e21800. doi:10.1371/journal.pone.0021800.

Swarbreck, D., Wilks, C., Lamesch, P., Berardini, T. Z., Garcia-Hernandez, M., Foerster, H., Li, D., Meyer, T., Muller, R., and Ploetz, L. (2008). The Arabidopsis Information Resource (TAIR): gene structure and function annotation. *Nucleic Acids Res.* 36, D1009–D1014. doi:10.1093/nar/gkm965.

Tang, H., Krishnakumar, V., Bidwell, S., Rosen, B., Chan, A., Zhou, S., Gentzbittel, L., Childs, K. L., Yandell, M., and Gundlach, H. (2014). An improved genome release (version Mt4. 0) for the model legume Medicago truncatula. *BMC Genomics* 15, 312. doi:10.1186/1471-2164-15-312.

Trapnell, C., Hendrickson, D. G., Sauvageau, M., Goff, L., Rinn, J. L., and Pachter, L. (2012a). Differential analysis of gene regulation at transcript resolution with RNA-seq. *Nat. Biotechnol.* 31, 46–53. doi:10.1038/nbt.2450.

Trapnell, C., Roberts, A., Goff, L., Pertea, G., Kim, D., Kelley, D. R., Pimentel, H., Salzberg, S. L., Rinn, J. L., and Pachter, L. (2012b). Differential gene and transcript expression analysis of RNA-seq experiments with TopHat and Cufflinks. *Nat. Protoc.* 7, 562–578. doi:10.1038/nprot.2012.016.

Tuskan, G. A., Difazio, S., Jansson, S., Bohlmann, J., Grigoriev, I., Hellsten, U., Putnam, N., Ralph, S., Rombauts, S., and Salamov, A. (2006). The genome of black cottonwood, Populus trichocarpa (Torr. & Gray). *Science.* 313, 1596–1604. doi:10.1126/science.1128691.

Venturini, L., Ferrarini, A., Zenoni, S., Tornielli, G. B., Fasoli, M., Dal Santo, S., Minio, A., Buson, G., Tononi, P., and Zago, E. D. (2013). De novo transcriptome characterization of Vitis vinifera cv. Corvina unveils varietal diversity. *BMC Genomics* 14, 41. doi:10.1186/1471-2164-14-41.

Wu, T. D., and Nacu, S. (2010). Fast and SNP-tolerant detection of complex variants and splicing in short reads. *Bioinformatics* 26, 873–881. doi:10.1093/bioinformatics/btq057.

Wu, T. D., and Watanabe, C. K. (2005). GMAP: a genomic mapping and alignment program for mRNA and EST sequences. *Bioinformatics* 21, 1859–1875. doi:10.1093/bioinformatics/bti310.

Young, N. D., Debelle, F., Oldroyd, G. E. D., Geurts, R., Cannon, S. B., Udvardi, M. K., Benedito, V. A., Mayer, K. F. X., Gouzy, J., Schoof, H., et al. (2011). The Medicago genome provides insight into the evolution of rhizobial symbioses. *Nature* 480, 520–524. doi:10.1038/Nature10625.
