# Supplementary material for: Genome-Wide Identification of Evolutionarily Conserved Alternative Splicing Events in Flowering Plants
Source: Front Bioeng Biotechnol. 2015 Mar 26;3:33. doi: 10.3389/fbioe.2015.00033 (PMC4374538; doi:10.3389/fbioe.2015.00033)
Supplement: Supplementary file 1 [file Data_Sheet_1.ZIP › Supplemental_Data_3 README_AS_EVENTS.docx]

“**Nine_Angiosperms_AS_Events.txt**” - is a tab-delimited file containing AS events in genes belonging to all nine angiosperms in this study. Below figure illustrates the mapping of genomic coordinates listed in the table.


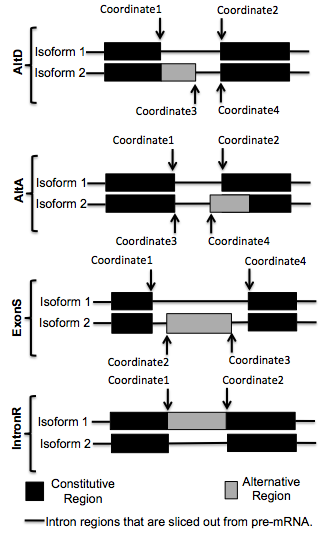


**The four types of AS events examined in this study**: AltD (alternative donor site), AltA (alternative acceptor site), ExonS (exon skipping), and IntronR (intron retention)

**Below are the species prefixes of AS genes listed in this file**: AmTr (*Amborella trichopoda*), Atha (*Arabidopsis thaliana*), Glyma (*Glycine max*), Medtr (*Medicago truncatula*), LOC (*Oryza sativa*), Phvul (*Phaseolus vulgaris*), Potri (*Populus trichocarpa*), Solyc (*Solanum lycopersicum*), and GSVIV (*Vitis vinifera*).
